# Curcumin as a Potential Antioxidant in Stress Regulation of Terrestrial, Avian, and Aquatic Animals: A Review

**DOI:** 10.3390/antiox12091700

**Published:** 2023-08-31

**Authors:** Do Thi Cat Tuong, Mohammad Moniruzzaman, Elena Smirnova, Sungyeon Chin, Anjana Sureshbabu, Adhimoolam Karthikeyan, Taesun Min

**Affiliations:** 1 Department of Animal Biotechnology, Jeju International Animal Research Center (JIA), Sustainable Agriculture Research Institute (SARI), Jeju National University, Jeju 63243, Republic of Korea; cattuongdothi@stu.jejunu.ac.kr (D.T.C.T.); elenas@stu.jejunu.ac.kr (E.S.); syeun77777@naver.com (S.C.); anjbio9@stu.jejunu.ac.kr (A.S.); 2 Subtropical Horticulture Research Institute, Jeju National University, Jeju 63243, Republic of Korea; karthik2373@gmail.com; 3 Department of Animal Biotechnology, Bio-Resources Computing Research Center, Sustainable Agriculture Research Institute (SARI), Jeju National University, Jeju 63243, Republic of Korea

**Keywords:** curcumin, antioxidants, terrestrial, avian, aquatic animals, stress management

## Abstract

Stress has brought about a variety of harmful impacts on different animals, leading to difficulties in the management of animal husbandry and aquaculture. Curcumin has been recognized as a potential component to ameliorate the adverse influence of animal stress induced by toxicity, inflammation, diseases, thermal effect, and so on. In detail, this compound is known to offer various outstanding functions, including antibacterial properties, antioxidant effects, immune response recovery, and behavioral restoration of animals under stress conditions. However, curcumin still has some limitations, owing to its low bioavailability. This review summarizes the latest updates on the regulatory effects of curcumin in terms of stress management in terrestrial, avian, and aquatic animals.

## 1. Introduction

Stress management in the animal husbandry and aquaculture industries has been under serious consideration in recent times. Animal stress can arise from various factors, such as extreme cold or heat in the climate, insufficient nutrition (including lack of food or water), physiological disorders, and disease outbreaks [1]. These stressors have had a wide range of impacts on animals in general and livestock, poultry, and fisheries in particular, including effects on growth, production, reproduction, and disease susceptibility [2]. For instance, heat stress is one of the contributing factors to reduced milk yields and quality, decreased meat production, and fertility issues in farm animals [3]. Moreover, thermal stress also induces physiological, behavioral, and production changes in poultry [4], as well as alterations in growth rate, oxidative stress, and immune response in fish [5]. As a result, animal stress has led to significant drawbacks in the agriculture sector and the nation’s economy. Thus, it is imperative to propose measures to address this issue. One potential solution that has garnered significant attention from scientists worldwide is the use of curcumin supplements to alleviate the deleterious effects of stress.

Curcumin is a natural biocomponent of turmeric (*Curcuma longa* L.) and has been used to treat inflammatory diseases, tumors, injuries, and so on, thanks to its anti-inflammatory, antioxidant, antibacterial, antifungal, and anticancer properties [6]. Curcumin has shown promise in mitigating the adverse effects of combatting neurodegenerative disorders, making it effective in the treatment of Alzheimer’s disease [7]. Moreover, curcumin has been found to prevent organ dysfunction and is recommended as a dietary supplement for obese individuals [8]. Related to counteracting the oncogenic stress management system, curcumin not only causes the death of different Kaposi’s sarcoma-associated herpes virus KSHV-positive cells but also obstructs the development of KSHV-infected cells [9]. On the other hand, several studies have been conducted on the role of curcumin in stress management in animal husbandry and aquaculture. Dietary curcumin supplementation has been found to benefit the growth performance of fish and young pigs [10]; enhance antioxidant status, nutrient absorption, thermotolerance and intestinal morphology in broiler chickens [11]; and improve nonspecific immune responses and stress resistance in the juvenile of greater amberjack [12].

Therefore, the following review aims to highlight the most significant effects of curcumin on stress in terrestrial animals, birds, and aquatic animals, especially fisheries, based on recent studies. By comprehending the potential benefits of curcumin supplementation in animals, researchers and veterinarians could explore its applications in diverse fields, such as livestock farming, aquaculture, animal welfare, and conservation. Moreover, additional investigation and experimentation are necessary to validate these findings in various animal species and contexts.

## 2. Overview of Curcumin

### 2.1. Biological Activities of Curcumin

**Figure 1:**
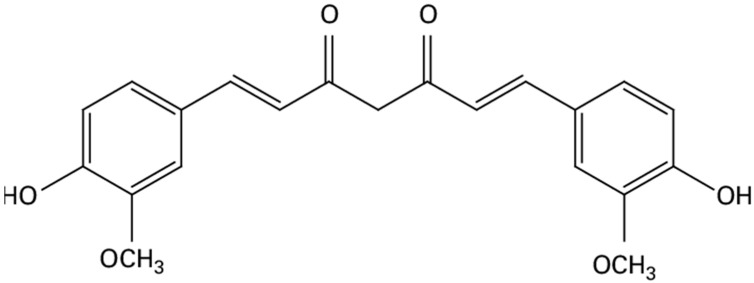
Chemical formula of curcumin.

Curcumin is a natural compound derived from the turmeric plant (*Curcuma longa*). Its chemical formula is C_21_H_20_O_6_ (Figure 1), and it has a molecular weight of 368.38 g/mol. Curcumin is known for its limited solubility in water, but it is freely soluble in various organic solvents, such as DMSO, ethanol, methanol, and acetone [13]. 

**Figure 2:**
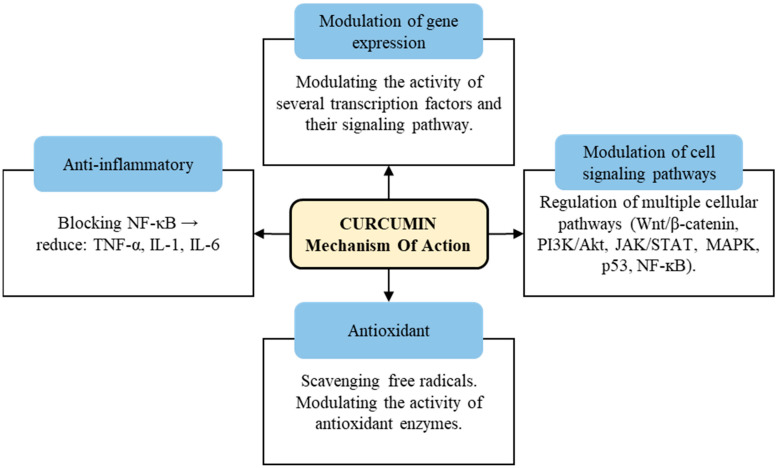
Schematic presentation on mechanism of action of curcumin.

Curcumin also has been extensively studied for its potential biological activities and health benefits. Although curcumin’s effects are diverse and complex, some of its most notable biological activities include antimicrobial activity, anti-inflammatory activity, antioxidant activity and antinociceptive activity, in addition to serving as a wound-healing agent [14]. The mechanism of action of curcumin involves multiple pathways and molecular targets (Figure 2) [15,16,17].

### 2.2. Metabolism of Curcumin

After being ingested, curcumin undergoes a sequence of metabolic transformations within the liver and intestines during phases I and II of metabolism. In phase I, reductase enzymes work to diminish the double bonds present in curcumin, leading to the creation of several metabolites, including octahydrocurcumin, dihydrocurcumin, hexahydrocurcumin, and tetrahydrocurcumin. Subsequently, both curcumin and its phase I metabolites progress to phase II metabolism, which involves conjugation with sulfate and glucuronic acid. This conjugation process gives rise to curcumin sulfates and curcumin glucuronides, respectively [18,19]. Following this, these metabolites circulate within the bloodstream and disperse throughout various organs in the body [19]. Among these, curcumin glucuronide appears to exhibit less bioactivity compared to curcumin and other metabolites [20]. Therefore, these metabolic transformations within the digestive system may play a role in limiting the oral bioavailability of curcumin [19]. It is essential to note that the bioavailability and metabolism of curcumin and its metabolites can vary among individuals due to factors such as genetics, gut microbiota composition, and the specific formulation of curcumin that is ingested.

### 2.3. Other Extractable Components of Turmeric

Apart from curcumin, turmeric, the primary curcuminoid, also contains two other curcuminoids: demethoxycurcumin and bisdemethoxycurcumin. These two analogs have a lower abundance of turmeric and exhibit weaker radical scavenging abilities than curcumin [21]. However, due to the high cost associated with extracting these curcuminoids individually from curcumin, they are often used in combination, yielding favorable outcomes. For instance, supplementation with a curcumin tablet (comprising 90% curcumin, 8% demethoxycurcumin, and 2% bisdemethoxycurcumin) for one week was reported to enhance vitamin C and E levels while also reducing malonaldehyde (MDA) and 8-hydroxydeoxyguanosine (8-OHdG) in the serum of precancerous patients [22]. Moreover, bisacurone, a sesquiterpene present in turmeric, also displays potential antioxidant properties. Bisacurone contributes to the reduction of triglycerides, cholesterol, and total lipid content, along with promoting lipolysis, making it a valuable component for preventing hepatic lipid accumulation [23]. 

### 2.4. Nanoformulation of Curcumin

As mentioned earlier, curcumin is not highly water-soluble and exhibits low bioavailability. This implies that a significant portion of ingested curcumin might not be effectively absorbed into the bloodstream through the gastrointestinal tract. To enhance absorption, several methods have been employed, such as combining curcumin with other compounds [18] or devising nanoformulations of curcumin [24]. Nanoparticles possess dimensions in the nanometer range (1~100 nm), which can enhance solubility, impede rapid metabolism, and enable targeted delivery to specific tissues or cells [25]. Notably, the use of nanoparticles resulted in the significantly higher bioavailability of curcumin glucuronide in plasma [20]. 

In addition to the approaches mentioned above, nanoformulations containing amphiphilic compounds (surfactants) have emerged as a potential strategy to ameliorate curcumin’s bioavailability. More specifically, curcumin-load solid lipid nanoparticle (SLNs) have demonstrated the ability to reduce particle size, leading to an enhanced anticancer effect [26]. 

## 3. Stress in Animals

**Figure 3:**
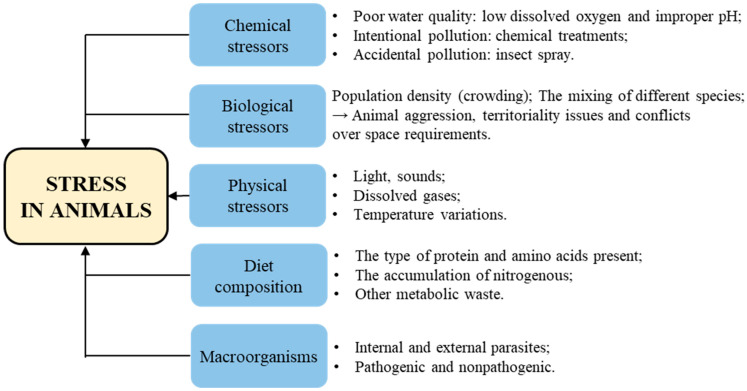
The causes of stress in animals.

Stress encompasses the entirety of biological reactions to physical, emotional, or mental stimuli that disrupt an individual’s homeostasis. The causes and mechanisms of stress in animals can differ depending on the species and their specific environments. A stressor can be defined as any internal or external stimuli or threat that disrupts the body’s homeostasis and elicits a coordinated physiological response in an attempt to restore balance. There are different types of stressors that can intensify stress, including chemical stressors, biological stressors and physical stressors. Additionally, factors such as macroorganisms and diet composition can also contribute to stress levels [27] (Figure 3).

The impacts of stress on animals can be significant and wide-ranging. When animals experience stress, it can affect their overall health in various ways. For instance, stress can suppress immune function, making animals more susceptible to diseases. It can also cause metabolic and hormonal changes, as well as decrease feed intake [27]. Furthermore, stressors can have implications for animal productivity, leading to a decline in protein and fat accretion in animal productions such as meat, eggs, and milk. This can compromise product quality and ultimately decrease product potential [28]. 

## 4. Curcumin in Stress Management of Terrestrial Animals

### 4.1. Oxidative Stress Management

Oxidative stress is a state in which the body is exposed to toxic reactive oxygen species (ROS) that overpower the antioxidant systems, resulting in an imbalance between them. This type of stress is considered harmful due to the damage caused by oxygen free radicals to adipose tissue, proteins, and DNA. Furthermore, oxidative stress is associated with various diseases, including atherosclerosis, hypertension, diabetes mellitus, ischemic diseases, and malignancies [29]. Hence, the utilization of antioxidant compounds such as curcumin in managing stress within the livestock industry has become increasingly important in recent times.

In the first place, oxidative stress can occur due to various reasons, one of which is toxicity resulting from toxins or heavy metals present in food sources or as side effects of drugs (Table 1). Aflatoxin B1 (AFB1), a potent mycotoxin, has significant impacts on human and animal health, including carcinogenic, mutagenic, teratogenic, and immunosuppressive effects [30]. AFB1 also induces oxidative stress by generating high levels of free radicals [31]. In a study conducted by Atef et al. [32], it was reported that curcumin has the ability to reduce hepatic oxidative stress in rabbits exposed to AFB1. This was evidenced by an increase in the ratio of antioxidant enzymes such as glutathione (SGH), catalase (CAT), and superoxide dismutase (SOD) and the removal of free radicals. However, it should be noted that curcumin can also have a genotoxic effect depending on its concentration [32]. Similarly, orally administered curcumin in mice showed protective effects against the detrimental influence of AFB1 on the kidneys. It improved renal antioxidant capacity while attenuating levels of blood urea nitrogen (BUN), uric acid, and creatinine [33,34]. Furthermore, another toxic chemical widely used in breeding to control pests is cypermethrin (CPM). It has various health effects, including neurotoxicity, reproductive toxicity, and molecular toxicity. CPM leads to a significant increase in MDA and protein carbonyl (PC) levels in serum, the liver, and the brain, while antioxidant biomarkers are significantly reduced in serum and brain tissues. Ziada et al. [35] indicated that plant phytochemicals such as vitamin C and curcumin play a protective role against cypermethrin-induced oxidative stress in serum, the brain, and the liver. Curcumin, in addition to its antioxidative and free-radical-scavenging abilities, also enhances the activity of other antioxidants [35]. Heavy metals such as cadmium (Cd) can cause damage to various organ systems in animals after long-term exposure. For instance, in Kunming mice, Cd significantly decreases semen quality, serum testosterone concentration, and the number of spermatogenic cells and mature spermatozoa. However, curcumin intervention improved these oxidative damages by activating the Nrf2 signaling pathway [36]. Similarly, the treatment of rats with nickel nanoparticles (NiNPs) at a concentration of 50 mg/kg for 28 days induces liver damage, but curcumin (at doses of 150 mg/kg or 300 mg/kg) can counteract this toxic effect, as evidenced by the reduction of alanine aminotransferase (ALT), aspartate aminotransferase (AST), and BUN levels in serum [37]. Moreover, the ingestion of curcumin significantly improved arsenic-induced hepatotoxicity and nephrotoxicity in male rats [38]. Apart from toxins, the side effects of drugs can also pose dangers to animals’ health. For example, although Ivermectin has many positive impacts in the treatment of sarcoptic mange, it has some side effects. In this case, turmeric extract was investigated and found to reduce the negative influences of Ivermectin-treated rabbits, while also promoting their performance, blood characteristics, and antioxidant status [39]. Likewise, ferrous ascorbate (FeAA) is a pro-oxidant that not only impairs motility and mitochondrial activity but also leads to a significant overgeneration of free radicals. Tvrdá et al. demonstrated that curcumin has a beneficial effect on bull spermatozoa under FeAA-induced oxidative stress. Particularly, at concentrations ranging from 25 to 50 µmol/L, curcumin has a profound impact on protecting these cells from free-radical-induced damage [40]. In addition, macrophages (RAW264.7 cells), which are sensitive to ROS, can be shielded from oxidative damage caused by H_2_O_2_ through the Nrf2-Keap1 pathway [41]. Moreover, oxidative stress can occur during the immune response process due to inflammation and diseases (Table 1). For instance, mastitis is a common disease in cattle breeding that reduces productivity and fertility. Medicinal plants, including *Curcuma longa*, have been extensively studied for their potential in treating this disease. The hexanic and ethanolic extracts of *C. longa* have shown anti-inflammatory properties against lipopolysaccharide (LPS)-induced inflammation. These extracts downregulate the expression of proinflammatory cytokines and exhibit free-radical-scavenging properties in buffalo mammary epithelial cells (BuMECs). They also upregulate the mRNA expression of the Nrf2 gene, which is involved in antioxidant defense mechanisms [42]. In the same way, nanoparticle-encapsulated curcumin has been found to have a beneficial influence on mastitis in mice, demonstrating superior effects compared to free curcumin, as shown in the study conducted by Suresh et al. [43]. Turmeric extract has also been shown to reduce the incidence and mortality rate of bovine respiratory disease (BRD) in crossbreed calves [44]. Furthermore, high-concentration curcumin and a mixture of piperine and curcumin have been shown to improve intestinal integrity and enhance antioxidant capacity, thereby reducing oxidative stress in weaned piglets [45]. Nevertheless, a study on the protective impact of curcumin on buffalo granulosa cells (GCs) revealed a negative effect on in vitro cultured cells when supplemented at higher concentrations and for longer durations. Specifically, cell viability and antioxidant enzyme activity were significantly decreased after 48 h of curcumin treatment [46]. Otherwise, excessive physical activities can lead to oxidative stress in animals (Table 1). While physical activities offer various benefits, overexercising can have certain unavoidable drawbacks, including liver and kidney injuries and tissue damage. In particular, the AST value is higher in exhaustion-exercise rats compared to non-exercise groups. To address these issues, the inclusion of antioxidants such as curcumin in the diet has been considered. Conceição et al. suggested that a combination of whey protein concentrate and curcumin could potentially decrease the expression of inflammatory cytokines and increase that of anti-inflammatory cytokines [47]. Furthermore, a mixture of cinnamon and turmeric has been shown to maintain and enhance antioxidant enzyme activities. This dietary supplementation has been found to affect growth parameters without impacting metabolism or damaging cellular machinery [48]. 

### 4.2. Thermal Stress Management

It is a well-known fact that global climate change intensifies the impact of temperature on livestock, leading to thermal stress that affects various health aspects, such as growth, physiological condition, immune function, morphology, and the antioxidant system in the body [49]. Thermal stress can be categorized into two types: cold stress and heat stress [50]. 

Heat stress occurs when an animal’s body temperature exceeds its thermoneutral zone due to high ambient temperature, solar radiation, and wind speed [51]. Prolonged exposure to these conditions can lead to hyperthermia, hyperventilation, endocrine changes, anorexia, and poor growth performance [52]. In such cases, in addition to providing thermal-controlled housing facilities, dietary interventions play a crucial role in mitigating the adverse effects of heat stress. Curcumin, an antioxidant and anti-inflammatory compound, has the potential to be used as a dietary supplement for animals experiencing heat stress.

In detail, as illustrated in a study by El-Ratel et al., the dietary intervention with curcumin or nanocurcumin had a significant positive impact on cecal activity, hematological parameters, and the reduction of harmful bacteria in heat-stressed rabbits, without any adverse effects on carcass traits [53]. Similarly, another research study showed that the use of turmeric extract reduced ear temperature and MDA levels, while increasing total antioxidant capacity in heat-stressed male rabbits, leading to improvements in heat tolerance, semen characteristics, and overall health [54]. In the same way, the addition of curcumin to the diet of female rabbits during the summer season in Egypt resulted in improved productive and reproductive parameters [55] (Table 2).

Another study indicated that the rectal temperature, ALT, AST, and serum lactate dehydrogenase (LDH) levels in mice subjected to high-temperature treatment were significantly elevated compared to those in the non-heat-stress group. However, dietary supplementation with curcumin was found to improve physiological stress and mitigate cardiac damage induced by heat treatment. This was achieved through the activation of antioxidant-related enzymes and the alleviation of physiological disorders [56]. Meanwhile, Zhao et al. [57] observed that curcumin significantly reduced the concentration of creatinine and blood urea nitrogen in serum, thereby preventing acute kidney injury in heatstroke rats. Apart from the effects on various organs of the body, during heat stress, albino rats exhibited restlessness, hypoactivity, a depressed attitude, and altered behavior. In contrast, rats treated with curcumin showed better behavioral changes after the heat-stress period, with moderate changes and a faster recovery time [58]. 

Additionally, the impact of the mixture of garlic, ginger, and turmeric on the biochemical parameters, immune function, and oxidative status of Damascus goats under heat stress conditions has been demonstrated [59]. Moreover, in Southern China, high temperature and humidity during the summer can have harmful effects on the growth and fertility of animals, including Hu Sheep, a local breed known for its high reproductive characteristics. In this context, curcumin has been found to have beneficial effects on the antioxidant status, immune ability, and reproductive performance of these sheep. This was evidenced by an increase in the activity of antioxidant enzymes and the concentrations of immunoglobulins and testosterone in the plasma of Hu Sheep [60]. 

Moreover, Grewal et al. [61] demonstrated that in vitro heat shock in buffalo mammary epithelial cells (BuMECs) led to distorted morphology, abnormal cell shape, and loss of cell–cell contact, along with a decrease in cell viability and proliferation. To address this issue, curcumin was added at different concentrations. Interestingly, the results showed that lower concentrations of curcumin (5 and 10 μM) provided better alleviation of the harmful effects of heat stress on the epithelial cells compared to higher-concentration groups. This was evidenced by the enhanced expression of heat-shock proteins, antioxidant genes, and anti-apoptotic genes [61] (Table 2).

Besides heat stress, low-temperature conditions also have negative impacts on livestock health and performance. An animal suffers cold stress when the temperature declines below the lower limit of the thermoneutral zone [62], and this situation leads to unavoidable consequences, such as a negative effect on calves’ weaning weight [63], spontaneous movements, exploratory behaviors, anxiety emotion in mice [64], an exerted oxidative stress hazard on sperm [65], and so on. Hence, measures to prevent cold stress in livestock should be seriously considered. Among these measures, curcumin supplementation is a safe and nontoxic solution, making it more attractive to scientists compared to other phytogenic compounds [66]. Turmeric-powder addition has been shown to enhance nutrient utilization in female calves during the winter season [67]. Furthermore, Hameed et al. [65] indicated that turmeric extract (100 µL/5 mL and 200 µL/5 mL) could improve the quality of cooled and post-thawed cattle bull semen. Likewise, 10 µM of curcumin had cryoprotective effects on Hariana bull semen by reducing protein carbonyls after freezing-thawing [68]. In goat semen cryopreservation, the curcumin nanoformulation (100 µg) was more effective in improving antioxidant status and sperm parameters compared to the nanoformulations of mint and thyme [69]. As for rabbits, the addition of curcumin and its nanoparticle improved the viability, progressive motility, and sperm ultrastructure of post-thawed semen through redox signaling and a reduction in the apoptosis process. The dose of 1.5 µg/mL curcumin nanoparticle yielded the best results [70].

**Table 2:** Regulatory effects of curcumin on thermal stress in terrestrial animals.

Animal Category	Experimental Design	Findings (Comparison to Negative Control)	Source
Heat stress			
APRI-line growing/weaned rabbits, aged 5 weeks, weighed 627.11 ± 2.51 g	A total of 100 rabbits were divided into 5 groups: G1 (control), G2 (CUR 20 mg/kg diet), G3 (CUR 25 mg/kg diet), G4 (nanoCUR 2.5 mg/kg diet), and G5 (nanoCUR 5 mg/kg diet). During growing period (8 weeks), ambient temperature relative humidity and temperature–humidity index were 32.77 °C, 43.23%, and 29.54, in turns.	↔carcass traits. ↔meat composition (moisture, crude protein). ↔Ph values of stomach, intestine, caecum. Caecum activity: ↑NH_3_-N, ↑VFAs. ↓harmful bacteria, ↓*E. coli*. Blood hematological parameters: ↑RBCs, ↓WBCs, ↓platelets, ↑HTC (G4, G5) ↔Hb, ↔erythrocytic indices (MCV, MCH, MCHC).	[53]
Mature rabbits, aged 6–7 months	A total of 70 male rabbits were divided into 7 groups: G1 (control_CD), G2 (CD + 30 mg/kg diet turmeric), G3 (CD + 60 mg/kg diet turmeric), G4 (CD + 90 mg/kg diet turmeric), G5 (CD + 50 mg/kg diet garlic), G6 (CD + 75 mg/kg diet garlic), and G7 (CD + 100 mg/kg diet garlic). Temperature: 30.45 °C ± 0.32 °C (max) and 26.24 °C ± 0.51 °C (min). Humidity: 75.35% ± 0.64% (max) and 52.10% ± 1.63% (min). The form of turmeric and garlic are in powder form.	↔final bodyweight, ↔feed intake ↓respiration rate, ↓ear temperature Hematological parameters: ↑Hb, ↑RBCs, ↑WBCs, ↑Platelets, ↑PVC. Serum antioxidants status: ↑TAC, ↓MDA, ↓total CHO, ↓triglyceride. Libido and semen characteristics: ↑mass motility, ↓dead sperm, ↑normal sperm, ↑TFSF, ↑MPS, ↓tail abnormality, ↑initial semen fructose. ↑relative epididymal weight, ↓germ cell apoptotic/seminiferous tubule, ↓relative weight of abdominal fat/kg, ↔relative testicular weight, ↔testicular measurements, ↔hepato-somatic, ↔renal-somatic, ↔spleen-somatic	[54]
New Zealand white (NZW) virgin female rabbits	A total of 45 healthy rabbits were divided into 3 groups: G1 (control), G2 (250 mg ginger powder), and G3 (250 mg CUR). The experiment was carried out during summer in Egypt. The powder of ginger/curcumin was mixed with a commercial pelleted diet.	↑CR, ↑kits born, ↑total kits at weaning, ↑litter size/individual (at birth and weaning), ↑average kit weight and litter weight/individual (at birth and weaning), ↑morality rate. ↑LBW, ↑FI, ↓water consumption. ↑albumin, globulin in blood. ↓urea, creatinine concentrations. ↓cortisol, ↑thyroid hormone (T_3_ and T_4_), ↑progesterone. ↓rectal, skin, ear temperature.	[55]
C57BL/6J mice, aged 6 weeks, weighed 18–20 g	A total of 48 mice were divided into 6 groups: G1 (no-heat treatment), G2 (HS), G3 (HS + ASA 1 mg/kg b.w.), G4 (HS + CUR 50 mg/kg b.w.), G5 (HS + CUR 100 mg/kg b.w.), G6 (HS + CUR 200 mg/kg b.w.). HS treatment: 41 °C for 20 min.	Indexed: ↓TMs, ↓BP, ↑HR. Serum biochemical parameters: ↓ALT, ↓AST, ↓LDH, ↔TP. Histological integrity: ↓myocardial fibers disorientation, ↓inflammatory cells. Biochemical markers: ↓cTn-I, ↓Ang II.	[56]
Sprague–Dawley (SD) rats, aged 65–70 days, weighed 190–220 g	A total of 50 rats were divided into 5 groups: G1 (NT control), G2 (DH control), G3 (CUR 50 mg/kg + DH), G4 (CUR 100 mg/kg + DH), and G5 (CUR 200 mg/kg + DH). CUR was dissolved in 0.5% CMCNa. DH: 41 ± 0.5 °C, 10 ± 1% humidity	↓creatinine, ↓BUN, ↓KIM-1, ↓NGAL. ↓expression of apoptosis-related proteins (Cyt-c, JNK, caspase-9): G4, G5.	[57]
Wistar-strain albino rats, weighed 150–180 g	A total of 24 animals were divided into 4 groups: G1 (DW), G2 (HS + DW), G3 (HS + CUR 0.5 g/kg), and G4 (HS + CUR 2.0 g/kg). CUR: powder (CUR-500™, >95% pure). HS: 37 ± 0.5 °C, 4 h/day	During heat stress: restlessness. After heat stress: - Activity level: G3 (hypoactivity), G4 (hypoactive initially only). - Attitude: G3 (depressed), G4 (near normal). - Provoked behavior: G3 (minimal response), G4 (moderate response).	[58]
Damascus goat bucks, aged 12–14 months, weighed 30 ± 1.23 kg	A total of 14 goats were divided into 3 groups: G1 (n = 4, control), G2 (n = 5, PHM 10 gm/head/day DM), and G3 (n = 5, QT 5 gm/head/day DM)	Sperm characteristics: ↑sperm concentration, ↑mass motility score, ↑sperm motility, ↑live spermatozoa, ↑normal spermatozoa, ↔[acrosomal integrity, normal sperm, primary, secondary sperm abnormalities, semen volume]. Hormone and blood biochemical constituents: ↑TST, ↔[TP, ALB, GLU, TG, AST], ↓ALT, ↓CHO. Antioxidant activities: ↓GPx, ↓MDA, ↓TCA. Hematological parameters: ↔[WBC, Hb, PCV, MCHC, MCH, MCV], ↑RBCs.	[59]
Hu sheep, aged 4 months, weighed 25.82 ± 0.34 kg	A total of 140 male Hu sheep were housed at temperature (33.32 ± 0.33 °C) and humidity level (70.56% ± 1.26%) and divided into 3 groups: G1 (control), G2 (CUR 450 mg/sheep), and G3 (CUR 900 mg/sheep).	Serum parameters: ↑NEFA (G3), ↔[GLU, TG, LDL, HDL, TC]. Antioxidant enzymes: ↔SOD, ↑GPx Plasma concentration: ↑[IgA, IgM, IgG] ↑TW/BW (G3), ↑TST, ↔testicular hsd3b mRNA. Apoptosis-related genes: ↓caspase-3 (G3)	[60]
Buffalo mammary epithelial cells (BuMECs)	Cells were cultured and divided into 7 groups: G1 (control); G2 (HT, 42 °C for 1 h); and G3–7 cultured with 5, 10, 20, 40, and 60 μM CUR, in turns, and then exposed to hyperthermia (42 °C for 1 h).	↓heat shock on the morphology Cell viability ↑[G3,G4], ↔[G5], ↓[G6,G7]. ↑antioxidant enzymes (SOD, CAT) [G3, G4]. Apoptosis-related genes: ↑BCL2 [G3, G4], ↓Bax, ↓caspase-3. Heat-shock protein: ↑HSP70 [G4], ↑HSP90 [G4, G5]. ↓Inflammatory-response-related genes (TNF-α, NF-κB) [G3, G4].	[61]
Cold Stress			
The female crossbred calves	A total of 24 female crossbred calves were divided into 4 groups: T1 (control), T2 (7.5 g garlic/head), T3 (7.5 g turmeric/head), and T4 (7.5 g garlic + 7.5 g turmeric/head). The experiment was carried out during winter season.	Growth performance, feed intake: highest value in T2. Nutrient utilization: ↑nutrient digestibility, ↓losses of nutrients.	[67]
Semen from five mature cattle bulls kept at a semen freezing center	Bull semen was divided into 4 groups: CON (control), TT1, TT2, and TT3 (turmeric extract 100, 200, and 300 µL/5 mL TCFY). Extend semen was cooled slowly to 5 °C and equilibrated for 2 h.	Cattle bull semen quality post-cooling: ↑motility, ↑alive, ↓abnormalities (TT1, TT2), ↑sperm membrane integrity, ↑acrosome (TT1, TT2). The post-thawed extended cattle bull semen: ↑motility, ↑alive, ↓abnormalities (TT2, TT3), ↑sperm membrane integrity, ↑acrosome (TT1, TT2). ↑in vivo fertility rate.	[65]
Healthy rabbit bucks, aged 10–12 months, weighed 3.6 ± 0.2 kg	Sperm cryopreservation of bucks was divided into 7 groups: control, CU0.5, CU1.0, and CU1.5 (0.5, 1.0, and 1.5 µg/mL CUR, respectively); CUNPs0.5, CUNPs1.0, and CUNPs1.5 (0.5, 1.0, and 1.5 µg/mL CUNPs, respectively).	Sperm characteristics (%): ↑progressive motility, ↑membrane integrity percentages, ↑viability (CU1.5, CUNPs), ↓abnormality. Sperm apoptosis (%): ↑viable, ↓early apoptosis, ↓late apoptosis, ↓necrosis. Antioxidants indices: ↑TAC, ↑SOD, ↑GPx, ↓MDA, ↓POC. Improve sperm ultrastructure.	[70]
Healthy Hariana bulls (*Bos indicus*), aged 7–8 years, weighed 450–550 kg	The diluted semen samples were divided into five aliquots: G1 (control), G2, G3, G4, and G5 (10, 25, 50, and 75 µM CUR, respectively). The temperature of semen straws reached from 4 °C to −140 °C within 7 min.	Functional sperm attributes: ↑the population (G4, G5), improve intact acrosome, intact membrane (G2, G3). Motility and kinematic: ↓the motile spermatozoa population (G4, G5), ↑total motility, progressive motility, and fast motility (G2, G3). Apoptotic- like changes: ↓DNA fragmentation, ↓deprotamination (G2). ↓carbonylated protein (G2). ↑Vanguard distance (G2, G3, G4).	[68]
Three sexually mature Baladi bucks, aged 2–4 years, weighed 50–60 kg	Semen samples were divided into 7 groups: G1 (control), G2 (MENFs 50 μg), G3 (MENFs 100 μg), G4 (TENFs 50 μg), G5 (TENFs 100 μg), G6 (CENFs 50 μg), and G7 (CENFs 100 μg). The diluted semen was cooled to 5 °C for 2 h.	Sperm quality in equilibrated semen: ↑[progressive motility, vitality, plasma membrane integrity] (CENFs). ↑post-thawing sperm quality. Sperm apoptosis and necrosis post-thawing: ↑viable spermatozoa, ↓apoptotic, ↓necrotic. Enzyme activity: ↔AST, ↔ALT. Extender post-thawing on oxidative stress: ↑TAC, ↓MDA.	[69]
Mature buffalo bulls	Semen from five bulls was divided into 5 groups: control, TTE1, TTE2, TTE3, and TTE4 (turmeric extract: 100 μL/5 mL, 200 μL/5 mL, 300 μL/5 mL, and 400 μL/5 mL TCFY, respectively). Extended semen was subjected to semen-freezing protocol.	Post-cooling: Sperm motility, alive sperms were significantly higher in TTE1. Sperm abnormalities lower in TTE1. Sperm membrane integrity was higher in TTE1. Acrosome percent was higher in TTE1, TTE2, TTE4. Post-thawing: Sperm motility was higher in TTE1. ↑Sperm membrane integrity (HOST). The conception rate was the best in TTE1.	[71]

Abbreviations: ↑(increase/upregulation), ↓(decrease/downregulation), ↔(no change), CUR (curcumin), CUNPs (nanoparticles curcumin), CD (commercial diet), GAR (garlic), NH_3_-N (ammonia nitrogen), VFAs (volatile fatty acids), RBCs (red blood cells), WBCs (white blood cells), MCV (mean corpuscular volume), MCH (mean corpuscular hemoglobin), MCHC (mean corpuscular hemoglobin concentration), Hb (hemoglobin), HTC (hematocrit), PVC (packed cell volume), TAC (total antioxidant capacity), MDA (malondialdehyde), TFSF (total functional sperm fraction), MPS (mitochondrial-potential sperm), PHM (plant herbs mixtures: garlic, ginger, and turmeric), QT (*Quebracho tannins* extract), TST (testosterone), TP (total proteins), ALB (albumin), GLU (glucose), CHO (cholesterol), TG (triglycerides), AST (aspartate aminotransferase), ALT (alanine aminotransferase), GPx (glutathione peroxidase), DW (distilled water), HS (heat stress), ASA (aspirin), TMs (rectal temperatures), BP (blood pressure), HR (heart rate), cTn-I (cardiac troponin I), Ang II (angiotensin II), NT (normal temperature), DH (dry heat), CR (conception rate), LBW (live bodyweight), FI (feed intake), LDL (low-density lipoprotein), HDL (high-density lipoprotein), TC (total cholesterol), POC (protein carbonyl), MENFs (mint nanoformulation), TENFs (thyme nanoformulation), CENFs (curcumin nanoformulation), CMCNa (sodium carboxymethyl cellulose), TCFY (whole egg yolk), CENFs (curcumin extract nanoformulations), MENFs (mint extract nanoformulations), TENFs (thyme extract nanoformulations) LDH (lactate dehydrogenase).

### 4.3. Nitrosative Stress Management

Similar to the imbalance between ROS and antioxidant systems in oxidative stress, the elevation of reactive nitrogen species (RNS) leads to nitrosative stress [72]. Ochratoxin A (OTA), a toxic secondary metabolite produced by the *Aspergillus* and *Penicillium genera*, adversely affects the reproduction, nutrition, and growth of animals. OTA induces overexpression of inducible nitric oxide synthase (iNOS), responsible for nitric oxide (NO) production. The excessive NO in the kidney and liver causes nitrosative stress, resulting in DNA damage and apoptosis. In contrast, curcumin treatment mitigates these harmful effects by reducing NO levels, iNOS, pro-inflammatory cytokines (NF-κB, TNF-α, IL-1β, and IL-6), and 8-OHdG, while regulating inflammation with fewer CD3+ T-lymphocytes in the tissues of OTA-poisoned rats [73]. Similarly, the ingestion of curcumin (50 mg/kg) and curcumin nanoemulsion (2.5 and 5 mg/kg) demonstrated comparable effects in male rats under protein-deficient conditions. Notably, curcumin in nanoemulsion form appears to be superior due to its nanometer size and higher solubility, leading to improved bioavailability [74].

## 5. Curcumin in Stress Management of Avian

### 5.1. Oxidative Stress Management in Birds

As can be seen, oxidative stress is one of the biggest reasons for the declined/compromised health and productive and reproductive performance of poultry. Overall, there are many oxidative stressors, such as temperature, toxins, microbial or virus challenges, and so on, and the antioxidant systems in living organisms have limited ability [75]. Thus, curcumin supplementation has been shown to be a safe and cost-effective solution.

To begin with, Aflatoxin B1 (AFB1), a mycotoxin produced by *A. flavus*, is a well-known cause of oxidative stress and damage in the liver [76,77,78], kidney [79], spleen [80], and duodenum [81] of poultry, which is highly sensitive to this toxin (Table 3). This toxin leads to an increase in free radicals, a decrease in antioxidant enzyme activity, inflammation, apoptosis in the liver of chickens and ducks, renal toxicity, abnormal functional and morphological changes in the duodenum of broilers, and lesions and immunotoxicity in the spleen of ducks. However, curcumin has shown the ability to restore the activity of antioxidant enzymes, serum antioxidant capacity, and NOX4 value and counteract DNA damage [79]. It also improves oxidative injuries, toxicity, inflammation, and apoptosis by regulating LncRNA-mRNA expression in broilers [76] and downregulating the expression of CYP450 enzymes, increasing ATPase activities and P-gp in chickens [81]. Similarly, curcumin positively influences oxidative stress and injuries in duck livers caused by AFB1 by improving lysosomal membrane permeabilization and lysosome biogenesis [77]. Moreover, curcumin supplementation activates the Nrf2 signaling pathway, preventing the expression of the NF-κB signaling pathway and downstream inflammatory factors, thereby decreasing the harmful impacts of AFB1 [80]. Apart from AFB1, fumonisin from *Fusarium* spp., mostly found in corn used in animal feed, also reduces bodyweight and induces necrosis of hepatocytes in chicks, even at low doses. Galli et al. [82] revealed that curcumin and its nanocapsules have hepatoprotective and antioxidant effects on these mycotoxin-consumed chicks. Interestingly, nanocurcumin at 10 mg/kg has superior protective and antioxidant effects compared to curcumin in its free state, as demonstrated by the reduction of thiobarbituric acid reactive substance (TBARS), ALT, AST, and ROS levels and the rise in SOD and CAT concentrations [82]. Likewise, Ochratoxin A (OTA) negatively impacts the growth and development of poultry, such as damaging lipid metabolism, disrupting cecum microbiota density, depressing antioxidative enzyme activities, reducing performance, and causing mitochondrial dysfunction. However, dietary curcumin can restore and enhance these functions in OTA-treated ducks. This is evidenced by the increase in CAT, SOD, total antioxidant capacity (T-AOC), and glutathione peroxidase (GSH-Px) levels, as well as the rehabilitation of richness indices and diversity indices in the composition of the intestinal microbiota [83,84].

Concerning heavy metals, arsenic trioxide (ATO), an environmental pollutant, was widely used in pesticides and insecticides. It can accumulate in water, soil, and plants, posing a long-term health risk to animals through consumption. Overconsumption of ATO can have serious consequences, including weight reduction, glomerular hemorrhage, mitophagy, apoptosis, and increased levels of ROS and MDA in the kidneys and skeletal muscle, leading to oxidative stress in ducks. As is evident from studies by Wu et al. and Lan et al., curcumin can improve growth speed, enhance renoprotective ability, and alleviate skeletal muscle injuries in ATO-infected ducks [85,86].

On the other hand, lipopolysaccharides (LPSs), components responsible for the pathological process of contamination induced by Gram-negative bacteria, can cause morphological damage to the ileum, lung injury, inflammation, and oxidative stress in poultry. The addition of 500 mg/kg of curcumin to the diet could ameliorate these symptoms in the lungs and ileitis of ducks through the signaling pathways of Nrf2-ARE, NF-κB, and TLR/NF-κB in an orderly manner [87,88]. Furthermore, curcumin has shown effectiveness against coccidiosis caused by Eimeria species. This is demonstrated by the improvement in antioxidant activities and lesion scores of the ceca, the reduction in oocyst shedding of *E. maxima* and *E. tenella* [89], and the decrease in enteric levels of 8-Iso-PGF2α [90]. 

Another factor that impacts broiler production is stocking density. Specifically, chickens stocked at high densities experience increased walking and standing behavior but decreased growth performance and antioxidant activities. In such oxidative stressful conditions, curcumin enhances the behavioral patterns, immune status, and growth performance of chicks. This is achieved by increasing immunoglobulin and antioxidant enzyme concentrations, while decreasing pro-inflammatory cytokine levels [91].

**Table 3:** Regulatory effects of curcumin on oxidative stress in birds.

Animal Category	Experimental Design	Findings (Comparison to Negative Control)	Source
commercial Arbor Acres (AA) broilers, aged one day old	A total of 32 broilers were divided into 4 groups: G1 (control group), G2 (1 mg/kg AFB1), G3 (1 mg/kg AFB1 + 300 mg/kg CUR), and G4 (300 mg/kg CUR).	Improving pathological live lesions. ↓[ALT, AST, AKP, γ-GT]. ↓[MDA, ROS]. ↑[GSH, CAT, SOD]. Normal cellular structure.	[76]
Ducks, aged one day old	A total of 60 ducks were divided into 3 groups: G1 (control), G2 (0.1 mg/kg AFB1), and G3 (0.1 mg/kg AFB1 + 400 mg/kg CUR).	↑SOD-1, ↑TRX, ↑HO-1, ↑Nrf2, ↓MDA. ↓P62, ↓LC3B, ↑mTOR, ↑ATG5, ↑LAMP1. ↓Gal3 protein, ↑CTSB.	[77]
Broiler chickens (Ross 308), aged 18 days, weighed 751.88 ± 46.28 g	A total of 32 male chickens were divided into 4 groups: G1 (control: BD), G2 (BD + 0.02 mg/kg feed AFB1), G3 (BD + 400 mg/kg feed CUR), and G4 (BD + 0.02 mg/kg feed AFB1 + 400 mg/kg feed CUR).	Enzyme activities: ↑SOD, ↑CAT, ↑GPx. ↑SAC, ↓MDA. Oxidative DNA Damage: ↓8-OHdG mRNA and protein expression: ↓mRNA. NOX4, ↓NOX4 (protein abundance).	[79]
Arbor Acres (AA) broilers, aged one day old	A total of 64 broilers were divided into 4 groups: G1 (control: basal diet), G2 (AFB1 5 mg/kg diet), G3 (AFB1 5 mg/kg diet + CUR 300 mg/kg diet), and G4 (CUR 300 mg/kg diet).	Serum enzyme activity: ↓ALT, ↓AST, ↓AKP, ↓GGT. Antioxidant enzymes activity: ↓MDA, ↑SOD, ↑CAT, ↑GSH. Oxidative stress marker (in serum and liver): ↓ROS, ↓8-OHdG. Histopathological observation: hepatic cords and cell structure recovery. The relative mRNA and protein expression: ↑Nrf2, ↑HO-1.	[78]
Ducks, aged one day old	A total of 60 ducks were divided into 3 groups: control, AFB1 (AFB1 0.1 mg/kg b.w.), CUR + AFB1 (AFB1 0.1 mg/kg b.w.+ CUR 400 mg/kg feed).	Spleen was smooth and uniform in color. Improve the damage to the spleen and the index of the spleen. Serum immunoglobulin content: ↓lgA, ↑IgG, ↑IgM. Histopathological alterations: ↑the number of ellipsoid lymphatic vessels and sheath-like capillaries, ↓the arterial wall thickening, ↑the count of lymphocytes, neutrophils. Inflammation-related genes: ↑the mRNA expression levels of NF-κB, IκB, TNF-α, IFN-γ, COX2, IL-1β, IL-2, IL-6, IL-18. ↓IL-4 mRNA expression levels. ↓the protein expression levels of p–NF–κB/NF-κB, p-IκB/IκB. ↓TNF-α. ↓p–NF–κB. ↓p-IκB. Nrf2 signaling pathway: ↑[Nrf2, HO-1, SOD-1, GPX2]mRNA expression, ↓keap1 (were returned to the same level as the control group).	[80]
Arbor Acres broilers, aged one day old	A total of 120 broilers were divided into 6 groups: C (control group), CC (CUR 450 mg/kg feed), L (AFB1 5 mg/kg + CUR 150 mg/kg feed), M (AFB1 5 mg/kg + CUR 300 mg/kg feed), H (AFB1 5 mg/kg + CUR 400 mg/kg feed), and AFB1 group (AFB1 5 mg/kg feed).	↓drowsiness, lethargy, and ruffled-feathers symptoms. Duodenum: ↓crypt depth, ↑villo height, ↑V/C. ↓SOD, ↓CAT, ↓8-OHdG ↑ATPase activities. ↓[CYP3A4, CYP2A6, CYP1A2, CYP1A1] ↑the expression of Abcb1 mRNA, P-gp.	[81]
Cobb-500 strain chicks, aged one day old	A total of 50 male chicks were divided into 5 groups: CP (positive control), CU (600 mg/kg fumonisin + 50 mg/kg CUR), NC5 (600 mg/kg fumonisin + 5 mg/kg nanoCUR), NC10 (600 mg/kg fumonisin + 10 mg/kg nanoCUR), and NC (negative control).	↑bodyweight (CU, NC10) Serum biochemistry: ↓glucose, ↓triglycerides (NC10), ↑cholesterol, ↓uric acid, ↓ALT (NC10), ↓AST. Oxidant and antioxidants profile: ↓TBARS, ↓ROS (NC10), ↑SOD, ↑CAT, ↓GST Necropsy and histopathology findings: liver (slightly yellow color), liver and intestines (no histopathological lesions).	[82]
White Pekin ducks, aged 1 day old, weighed 43.4 ± 0.1 g	A total of 720 mixed-sex ducks were divided into 4 groups: CON (control group), OTA (2 mg/kg OTA), CUR (400 mg/kg CUR), and OTA + CUR (2 mg/kg OTA + 400 mg/kg CUR).	Serum liver function: ↓AST, ↔[AST, TC, TG, HDL]. Antioxidative capacity: ↔T-AOC, ↑SOD, ↑CAT, ↔GSH-Px, ↓MDA. ↑ACE, ↑Simpson indexes. Recovered the microbiota composition. mRNA expressions: ↓FAS, ↑Nrf2, ↑HMOX1	[83]
White Pekin ducks, aged 1 day old, 43.4 ± 0.1 g	A total of 540 mixed-sex ducks were divided into 3 groups: G1 (control), G2 (2 mg/kg OTA), and G3 (2 mg/kg OTA + 400 mg/kg CUR).	↑growth performance Antioxidant parameters and jejunal cytokines: ↑GSH-Px, ↑SOD, ↑T-AOC, ↓IL-1β, ↑IL-10, ↓TNF-α, ↓DAO ↑villus height, ↓crypth depth The expression of genes related to apoptosis: ↑Bcl-2, ↓CASP3 Mitochondrial transcription factor: ↓TFAM, ↓TFB1M, ↓TFB2M.	[84]
Ducks, aged 1 day old	A total of 75 ducks were divided into 5 groups: CON (control group), LA (low-dose ATO group: 2 mg/kg ATO), MA (medium-dose ATO group: 4 mg/kg ATO), HA (high-dose ATO group: 8 mg/kg ATO), AC (8 mg/kg ATO + 400 mg/kg CUR feed).	↑bodyweight. ↓muscle arsenic concentration ↑T-AOC, ↓SOD, ↓MDA Improve mitochondrial structure. mRNA expression levels: ↑OPA1, ↑Mfn, ↓Drp1, ↑Nrf1, ↑Nrf2, ↑TFAM. Mitophagy: ↓PINK1, ↓Parkin, ↓LC3-I, ↓LC3-II, ↓p62 Mitochondria-mediated apoptosis: ↓p53, ↓Bax, ↑Bcl-2, ↓Cytc, ↓caspase-3.	[85]
Sansui white ducks, aged 1 day, weighed 50–100 g	A total of 32 ducks were 4 groups: G1 (control—deionized water), G2 (4 mg/kg ATO), G3 (8 mg/kg ATO), and G4 (8 mg/kg ATO + 400 mg/kg CUR).	↑Bodyweight (G4 ducks grew faster). ↓ATO levels in serum and kidney. ↓damage in kidney tissues. ↓Relative mRNA levels (Nrf2, GPX-1, CAT, SOD-1, HO-1). Protein expression levels: ↑Nrf2, ↓Trx, ↑SOD-1, ↓HO-1, ↑T-AOC, MDA. ↓autophagy-related mRNA and protein levels (mTOR, LC3-I, LC3-II, Atg-5, Beclin1, Pink, Parkin)↓apoptosis-related mRNA and protein expression levels (caspase-3, Cytc, p53, Bax).	[86]
Specific-pathogen-free *Anas platyrhynchos* ducks, aged 1 day old, weighed 34.00 ± 0.50 g	A total of 450 male ducks were divided into 3 groups: CON (control: basal diet), LPS (basal diet + LPS 5 mg/kg b.w.), and LPS + CUR (basal diet + LPS 5 mg/kg b.w. + CUR 500 mg/kg b.w.). CUR: powder form.	Repairing the inflammatory manifestation of ling tissues. Antioxidant capacity of the plasma: ↑GSH-Px, ↓MDA, ↑T-SOD. Expression of genes (Nrf2-ARE signaling pathway): ↑Nfr2, ↓Keap1, ↑CAT, ↑HO-1, ↑SOD-1, ↑GCLC, ↑GCLM, ↑NQO-1. Expression of genes (NF-κB signaling pathway): ↓[TLR4, NF-Κb, TNF-α, IL-6, IL-8, NLRP3, caspase-1.	[87]
Specific-pathogen-free (SPF) ducks (*Anas platyrhynchos*), aged 1 day old, weighed 35 ± 1 g	A total of 40 male ducks divided into 4 groups: C_0_ (corn–soybean basal diet), C_0_ + LPS (corn–soybean basal diet + 0.5 mg/kg b.w. LPS), C_500_ (0.5 g/kg b.w. CUR), and C_500_ + LPS (0.5 g/kg CUR + 0.5 mg/kg b.w. LPS).	Ileum morphology: ↓villus height, crypt depth (highest: C_0_ + LPS, lowest: C_500_ + LPS), ↑villus height/crypt depth. mRNA expression of antioxidant genes: ↑Nrf2, ↓Keap1, ↓SOD1 (C_0_ + LPS: highest), ↔CAT, HO-1 (C_500_ highest), ↔NQO-1, ↑GCLM, ↑GCLM. mRNA expression of inflammatory-related gene: ↑TLR4, ↑NF-κB, ↑TXNIP, ↑IL-1β, ↑IL-6, ↑TNF-α. Protein expression: ↑Nrf2, ↓HO-1, ↑TXNIP.	[88]
Cobb-500 breed broiler chicks, aged 12 days old	A total of 360 male chicks were divided into 6 groups: NCC, NCC + 100 mg/kg CUR, NCC + 200 mg/kg CUR, CC, CC + 100 mg/kg CUR, and CC + 200 mg/kg CUR.	↔growth parameters. Lesion score: ↔duodenal, ↔jejunum and ileum, ↓cecum. Intestinal permeability: ↓(CC + 100 mg/kg CUR), ↑(CC + 200 mg/kg CUR). Oocyst Shedding: ↓Count of *E. maxima*. Glutathione: [↑GSH, ↑GSSG, ↑total glutathione] CC + 100 mg/kg CUR.	[89]
Cobb-500 chicks, aged 2 weeks	A total of 200 birds were divided into 4 groups: G1 (MSD, negative control), G2 (HSD, positive control), G3 (HSD, CUR 100 mg/kg diet), and G4 (HSD, CUR 200 mg/kg diet). MSD (10 birds/m^2^) and HSD (20 birds/m^2^).	Productive performance: ↑bodyweight, ↑food intake, ↑feed conversion ratio. Behavioral observation: enhancement [ingestive behavior, crouching, body care behavior], ↓[walking, standing behavior]. Hematological parameters: ↑PVC, ↑Hb, ↑RBCs, ↔WBCs, ↓ERS, ↓H/L ratio. Immunological parameters: ↑IgG, ↑IgA, ↑IgM, ↓IL-2, ↓IL-6, ↓TNF-a. Hormonal analysis: ↓ALT, ↓AST, ↓total cholesterol. Antioxidant measurements: ↑[SOD, GPx, CAT], ↓MDA. Hormonal concentrations: ↑T3, ↑T4, ↓corticosterone. Gene expression: ↑[GHR, IGF-1]	[91]

Abbreviations: ↑(increase/upregulation), ↓(decrease/downregulation), ↔(no change), BD (basal diet), CUR (curcumin), SOD (superoxide dismutase), CAT (catalase), GPx (glutathione peroxidase), SAC (serum antioxidant capacity), MDA (malonaldehyde), 8-OHdG (8-hydroxy-2-deoxyguanosine), AFB1 (aflatoxin B1), ATO (arsenic trioxide), T-AOC (total antioxidant capacity), Nrf2 (the nuclear-factor-erythroid-2-related factor 2), GSH (glutathione), CAT (catalase), HO-1 (heme oxygenase), ROS (reactive oxygen species), MSD (low stocking density), HSD (high stocking density), PCV (packed cell volume), Hb (hemoglobin), RBCs (red blood cells), WBCs (white blood cells), ESR (erythrocyte sedimentation rate), H/L (heterophil/lymphocyte), AST (aspartate aminotransferase), ALT (alanine aminotransferase), AKP (alkaline phosphatase), GGT (gamma glutamyl transpeptidase), LAMP1 (a lysosomal membrane protein), CTSB (cathepsin B), GST (glutathione reductase), BW (bodyweight), LPS (lipopolysaccharides), OTA (ochratoxin A), DAO (diamine oxidase), NCC (nonchallenged control), CC (challenged control), TBARS (thiobarbituric acid reactive substances).

### 5.2. Thermal Stress Management in Birds

During the heat stress period, physiological, behavioral, and immunological abnormalities might occur, resulting in disadvantages for bird productivity [92]. Otherwise, these negative effects can be alleviated by supplementing with curcumin [93]. 

As an illustration, Salah et al. suggested that a concentration of 100 mg/kg of curcumin in the diet could ameliorate the average daily feed intake and the unsaturated fatty acids and enhance the levels of adenosine triphosphate (ATP) and Coenzyme Q10 (CoQ10) in liver tissue, as well as the brain serotonin of heat-stroke broilers [94]. Meanwhile, as is evident from the research of Mustafa et al. [95], the serum biochemical parameters of heat-stressed broiler chickens such as lipid, protein profile, creatine kinase, uric acid, and glucose have improved after the addition of curcumin or turmeric powder to the diet. In addition, turmeric (500 mg/kg bodyweight) was able to raise the quality of the chest and thigh meat and decrease the total cholesterol level of chickens reared in heat stress [96]. The supplementation of 0.2% dried turmeric rhizome powder has especially been proven to be better than betaine (Bet) for ameliorating humoral immunity and stress tolerance in heat-stressed broilers [97]. Conversely, 0.5% turmeric powder did not have any impact on growth and caused an enormous decline in the feed conversion ratio; however, it could improve thyroid hormones, increase feed intake, and reduce blood MDA and lipid peroxidation in the heat-stress broiler [98,99]. Moreover, the addition of turmeric root powder, as well as its mixture with carnation flowers, to the diet of broilers under heat stress conditions showed an increase in the percentage of lymphocytes and the concentration of total protein and globulin; meanwhile, while the levels of glucose and uric acid and the number of harmful bacteria were declined [100]. Correspondingly, the 8 g/kg diet of *C. longa* powder also resulted in better intestinal morphology under hot tropical environments, leading to the amelioration of nutrient absorption and thermotolerance of broiler chickens [11]. On the other hand, dietary curcumin might intensify antioxidant ability and immunity and ease the stress symptoms of laying hens under high-temperature environment conditions, as visualized by the activities of CAT, SOD, GSH-Px, and T-AOC in the liver, heart, and lung tissues for curcumin-treatment groups being higher, while the statistics for the corticosterone levels, ALT, and inflammatory-cytokine response were lower than that for control groups [101,102]. Furthermore, the supplementation of curcumin on chicken embryonic fibroblast cells (CEFs) could reduce ROS and MDA levels and reverse the downregulation of the expression of the antioxidant enzyme via the MAPK-Nrf2 signaling pathway under high-temperature conditions [103] (Table 4).

Among the environmental stressors, cold stress also poses several disadvantages to animals (Table 4). For example, it suppresses body temperature, oxygen consumption, and respiratory water loss [92]. A study on Ross-308 male broiler chicks revealed that cold-stressed birds had a lower weight gain but higher levels of MDA and AST compared to normal birds. However, the inclusion of curcumin and nanocurcumin in the diet proved effective in mitigating these issues. Notably, a dosage of 200 mg/kg curcumin exhibited the ability to reduce MDA and total cholesterol levels, while improving the immune system, microbial population, and liver enzyme activities. In contrast, in the same study, nanocurcumin demonstrated lesser positive impacts due to its higher concentration [104]. Furthermore, during the winter season in the southern region of Brazil, the egg quality and antioxidant capacity of Japanese quails and Hy-Line Brown laying hens were enhanced by incorporating curcumin into their diets [105,106].

**Table 4:** Regulatory effects of curcumin on thermal stress in birds.

Animal Category	Experimental Design	Findings (Comparison to Negative Control)	Source
Heat Stress			
Chicks (Ross strain), aged 120 days old	A total of 30 male chicks were divided into 3 groups: T1 (control), T2 (34 °C 8:00–16:00, basal diet), and T3 (34 °C 8:00–16:00, basal diet + CUR 100 mg/kg diet).	Improved the average daily feed intake. ↑Dressing percentage, ↑breast yield, ↓abdominal fat, ↔[leg, liver, heart]. Fatty Acid profile: ↑MUFAs (myristoleic, palmitoleic, oleic), ↑PUFAs (linoleic, docosahexaenoic, eicosapentaenoic), ↓saturated FAs in breast (myristic and palmitic) and thigh (palmitic and stearic) muscles. ↓MDA, ↑ATP, ↓ADP, ↑CoQ10, ↓Na, K-ATPase, ↑5HT, ↓5-HIAA.	[94]
Broiler chickens	A total of 100 chickens (maintained in heat stress) were divided into 3 groups: control, ascorbic acid group (dose: 60 mg/tail/day), and turmeric group (dose: 500 mg/kg bodyweight).	↔Broiler performance: bodyweight gain, feed conversion, feed efficiency. Quality of carcass: ↑carcass percentage, ↑percent of thigh meat, ↑percentage of breast meat, ↑liver weight, ↑gizzard weight. ↓cholesterol.	[96]
Ross-308 chicks, aged 50 days old	A total 700 chicks were placed in two halls: normal condition (N) and heat-stressed (S) condition (37 °C). Under each condition, chicks were divided into 5 groups: T1 (basal diet), T2 (CUR 50 g/ton feed), T3 (CUR 75 g/ton feed), T4 (turmeric powder 1.65 kg/ton feed), and T5 (turmeric powder 2.5 kg/ton feed).	Serum lipid profile: ↓cholesterol, ↑HDL, ↓LDL, ↓VLDL, ↓triglyceride. Serum protein profile: ↑albumen, ↑globulin, ↑total protein. Thyroid hormones: ↑T_3_, ↑T_4_. ↓ALT, AST enzymes. ↓serum creatin kinase, uric acid, glucose.	[95]
Cobb-500 broiler chicks, aged 31 days old	A total of 300 mixed-sex chicks were divided into 5 groups: A (control), B (0.5% turmeric powder), C (0.5% cinnamon powder), D (0.5% ginger powder), and E (0.5% garlic powder). During days 31–42 of the rearing period, the chicks were exposed to environmental temperature (32–34 °C) daily, from 12 a.m. to 16 p.m., to induce heat stress.	↓bodyweight, ↑average daily gain, ↑average daily feed intake, ↓feed conversion ratio, ↑rectal temperature mean (42-day-old chickens), ↓bursa of Fabricius weight (42-day-old chickens), ↑chickens spleen weight (42-day-old chickens), ↑SOD, ↑GPx, ↓CAT, ↓MDA, ↑total antioxidant capacity, ↑ALP, ↑CE, ↑T_3_, ↑T_4_.	[99]
Ross chicks, aged one day old	A total of 200 male chicks were divided into 4 groups: G1 (control), G2 (0.5% turmeric), G3 (0.5% cinnamon), and G4 (0.25% cinnamon + 0.25% turmeric). All birds were treated with heat stress (32 °C).	Performance: ↑feed intake, ↑feed intake, ↓feed conversion ratio. Blood, enzyme, and antioxidant parameters: ↓AST, ↓ALT, ↓LDH, ↓uric acid, ↔urea, ↑creatinine, ↓MDA. ↓chlorine, ↔sodium, ↑potassium, ↑hematocrit, ↔rectal temperature.	[98]
Ross-308 chicks, aged one day old	A total of 625 mixed-sex chicks were divided into 5 groups: TN-CON (thermoneutral), HS-CON (heat stress-control), HS-Bet (0.1% betaine), HS-TRP (0.2% turmeric rhizome powder), and HS-BT (0.1% betaine + 0.2% turmeric rhizome powder).	↑bodyweight gain, ↑feed intake, ↓feed-to- gain ratio. Blood leukocyte profile: ↓monocytes, ↓eosinophil, ↓basophils, ↓heterophil, ↑lymphocyte. Antibody titers against SRBC: ↑total antibody, ↑IgM, ↔IgG (28 days of age), ↑IgG (42 days of age). ↔TAC, ↓MDA, ↑GPx, ↑SOD.	[97]
Ross-308 chicks, one day old	A total 360 broiler chicks were divided into 6 groups: T0 (control), T1 (3 mg/kg diet of turmeric), T2 (5 mg/kg diet of turmeric), T3 (3 mg/kg diet of carnation flower powder), T4 (5 mg/kg diet of carnation flower powder), and T5 (4 mg/kg mix of turmeric and carnation flower).	↑WBC, ↓heterophil %, ↑lymphocytes %. The biochemical characteristics: ↓glucose, ↑total protein, ↔albums, ↑globulin, ↓uric acid. The number of bacteria: ↓*E. coli*, ↓Salmonella, ↑Lactobacillus.	[100]
Chick broilers (Marshal), aged one day old	A total 240 chicks were divided into 4 groups: CN (corn-soy based diet), FG (basal diet + 4 g/kg *C. longa* powder), EG (basal diet + 8 g/kg *C. longa* powder), and TT (basal diet + 12 g/kg *C. longa* powder).	The juvenile growth performance: ↑initial weight, ↑final weight, ↑weight gain, ↔feed intake. The villus height: ↑duodenum, ↑jejunum, ↑ileum. The villus width: ↑duodenum, ↑jejunum, ↑ileum. The crypt depth: ↑duodenum, ↑jejunum, ↑ileum. ↓The respiratory rate, ↓breast temperature, ↓comb temperature, ↔heart rate. The hematological parameters: ↔(PCV, hemoglobin, red blood cells, white blood cells, lymphocyte across). Physiological responses: ↓MDA, ↓rectal temperature, ↑T3, ↑uric acid.	[11]
Roman egg-laying hens, aged 22 weeks old, weighed 1420 g (start of experiment), aged 31 weeks old, weighed 1940 g (terminated)	A total of 336 hens were divided into 7 groups: TC (thermo-neutral control), HC (heat control), H1 (HC + 100 mg/kg CUR), H2 (HC + 150 mg/kg CUR), H3 (HC + 200 mg/kg CUR), H4 (HC + 250 mg/kg CUR), and H5 (HC + 300 mg/kg CUR).	Serum antioxidant metabolites: ↑SOD, ↑CAT, ↑T-AOC, ↑GSH-Px, ↓MDA. Antioxidant metabolites in liver tissue: ↑SOD, ↓CAT (H5, 6 weeks), ↓T-AOC (H5, 9 weeks), ↓GSH-Px (6 weeks), ↑MDA (H4, 6 weeks; H5, 9 weeks). Antioxidant metabolites in heart tissues: ↑SOD, ↓CAT (H1, 3 weeks), ↑T-AOC, ↓GSH-Px (H2, H5, 6 weeks), ↑MDA (H2, 6 weeks; H3, H4, H5, 9 weeks). Antioxidant metabolites in lung tissues: ↑SOD, ↓CAT (H1, 3 weeks), ↓T-AOC (H1, H4, H5, 3 weeks), ↓GSH-Px (6 weeks), ↓MDA (H3, H4, H5, 3 weeks; 6 weeks; H2, H3, H4, 9 weeks)	[101]
Roman egg laying hens, aged 25 weeks old	A total 250 hens were divided into 5 groups: NC (normal temperature control 22–25 °C), HC (high temperature 32 ± 1 °C), HT100 (HC + 100 mg/kg CUR), HT200 (HC + 200 mg/kg CUR), and HT300 (HC + 300 mg/kg CUR).	Corticosterone serum level: ↓(HT100, HT200), ↔HT300. WBC parameters: ↑HT200, ↔(HT100, HT300). Heterophil/lymphocyte (H/L) ratio: ↓(HT100, HT200), ↔HT300. Serum IgG and IgM: ↑(HT100, HT200), ↔HT300. Serum cytokines: ↓(IL-6, IL-1β, TNF-α) HT100, HT200.Liver enzymatic activity: ↓ALT (HT100, HT200).	[102]
Chicken embryonic fibroblast cells (CEFs)	A CEF cell line was divided into 6 groups: NC (normal temperature group) H (high-temperature control group), H1(5 μmol/L CUR), H2 (10 μmol/L CUR), H3 (20 μmol/L CUR), and H4 (40 μmol/L CUR).	Cell viability: ↑(H2, H3, H4) after 12 h; ↑H3_after 24 h. ↑cell proliferation. ↓cell apoptosis rate (H3, H4). ↓ROS (H3, H4), ↓MDA. Antioxidant enzyme activity: ↑CAT, ↑SOD, ↑GSH-Px. ↑genes expression (CAT, SOD1, SOD2, GSTO1, GSTT1, GSTA3). ↑MAPK- Nrf2 pathway genes (Nrf2, Jnk, Erk, P38).	[103]
Cold Stress			
Ross-308 broiler chicks, aged one day old	A total of 250 male chicks were divided into 5 groups: I (control), II (200 mg/kg CUR), III (400 mg/g CUR), IV (200 mg/g nanocurcumin), and V (400 mg/g nanocurcumin). First week: 32 °C. Second week: 29 °C. Thereafter, the temperature gradually dropped to 15 °C on day 14.	Performance: ↔feed intake, ↑feed conversion ratio (nanocurcumin). ↑weight gain (III, IV). Liver enzyme activities: ↓MDA, ↓LDH, ↓AST. Blood cholesterol: ↓total cholesterol, ↑HDL, ↓LDL, ↓triglycerides. Immuno-function: ↑WBC, ↓heterophils, ↑lymphocytes, ↓heterophils/lymphocytes ratio.	[104]
Japanese quails (*Coturnix japonica*), aged thirty days old	A total of 60 quails were divided into 4 groups: T0 (control), T30 (30 mg/kg diet free CUR), T3 (3 mg/kg diet nanocapsules CUR), and T10 (10 mg/kg diet nanocapsules CUR). The experiment was carried out in a shed, without air-conditioning, during the winter (1 °C–17 °C).	Performance: ↑egg production, ↑egg weight (T30), ↑egg mass, ↔feed intake, ↓feed conversion (g/g), ↓feed conversion (g/dozen_T10). Egg chemical composition: ↔(specific gravity, Haugh unity, yolk index, yolk pH, albumen pH, yolk percentage, eggshell percentage, albumen percentage); ↑(luminosity, yellow intensity). Oxidant/antioxidant status: ↓TBARS, ↑ACAP Fatty acid profile in egg: ↓SFA (T10), ↑MUFA, ↑PUFA (T10)	[105]
Hy-Line Brown laying hens, aged 84 weeks old, weighed 1680 ± 10 g	A total of 36 hens were naturally infect with *E. coli* and divided into 2 groups: T-CON (control) and T-CUR (CUR 200 mg/kg). The experiment was carried out in a shed, without air-conditioning, during the winter (−2.5 °C–19.7 °C).	Egg quality: ↔(Haugh units, albumen pH, yolk weight, egg shell strength, red intensity, shell thickness) in days 21 and 42. ↑(yellow and brightness in fresh eggs). ↓TBARS, ↑ACAP. Antioxidant status: ↓LPO, ↑GPx, ↑GST. ↔(red cell number, hematocrits, hemoglobin concentrations, eosinophil, monocyte). ↓(total protein, alkaline phosphatase, alanine aminotransferase), ↔(globulin, albumin levels). Fecal microbiology: ↓bacterial counts (coliforms and *E. coli*).	[106]

Abbreviations: ↑(increase/upregulation), ↓(decrease/downregulation), ↔(no change), CUR (curcumin), 5HT (serotonin), 5-HIAA (5-hydroxyindoleacetic acid), SOD (superoxide dismutase), CAT (catalase), GSH-Px (glutathione peroxidase), HBC (hemoglobin concentration), RBC (red blood cell), WBC (white blood cell), AST (aspartate aminotransferase), ALT (alanine aminotransferase), ALP (alkaline phosphatase), CE (corticosteroid), SRBC (sheep red blood cell), TAC (total antioxidant capacity), MDA (malondialdehyde), GPx (glutathione peroxidase), WBC (white blood cell), ACAP (Antioxidant capacity against peroxyl radicals), SFA (sum of saturated fatty acids); MUFA (sum of monounsaturated fatty acids), PUFA (sum of polyunsaturated fatty acids), ATP (adenosine triphosphate), ADP (adenosine triphosphate), CoQ10 (Coenzyme Q10), HDL (high-density lipoprotein), LDL (low-density lipoprotein), VLDL (very low density lipoprotein), LDH (lactate dehydrogenase), PCV (packed cell volume), T-AOC (total antioxidant capacity), TBARS (thiobarbituric acid reactive substances), LPO (lipid peroxidation).

## 6. Curcumin in Management of Aquatic Animals

### 6.1. Oxidative Stress Management in Aquatic Animals

According to numerous studies, oxidative stress in aquaculture can be induced by various chemotoxic agents (pesticides, insecticides, etc.) or environmental factors (DO, pH, salinity, etc.), which can impact various biological processes, including fish development, physiology, and metabolic processes [107]. Due to its safety, affordability, nontoxic nature, and remarkable functionality [66], curcumin offers a superior dietary supplement compared to other options for addressing this issue (Table 5).

Firstly, one of the most common stress factors in fisheries is related to toxicity and heavy metals. Hydrogen peroxide (H_2_O_2_), which is used as a disinfectant in aquaculture [108], also induces oxidative stress in aquatic animals. In this regard, Wang et al. [109] indicated that a concentration of 2–4 g/kg turmeric aqueous extract protected spotted seabass (*Lateolabrax maculatus*) from the harmful effects of H_2_O_2_ through the Nrf2/Keap1 pathway. In the same way, turmeric leaf extract (TLE), which contains curcumin, was found to reduce ROS generation, lipid peroxidation, and cell death in H_2_O_2_-treated zebrafish [110]. In terms of the harmful effects of heavy metals, Rajabiesterabadi et al. [111] reported that copper exposure caused inflammation, anemia, and hepatic damage in common carp (*Cyprinus carpio*), as evidenced by increased cortisol, MDA, ALT, and AST levels, while red blood cell count and hemoglobin decreased. The addition of 10 g/kg dietary turmeric is recommended to mitigate the negative impacts induced by copper by enhancing SOD, CAT, glutathione peroxidase (GPx) activity, and the expression of IL10 [111]. Melamine (MEL), which is widely used in crop fertilizers and pesticides, has several detrimental effects on the growth, immune response, disease resistance, and oxidative stress in various fish species. Abd El-Hakim et al. [112] found that a dosage of 200 mg/kg curcumin improved bodyweight and hematological variables, minimized reductions in lysozyme activity and total protein, and enhanced oxidative stress indices in MEL-treated Nile tilapia (*Oreochromis niloticus*). Likewise, chlorpyrifos (CPF), a broad-spectrum organophosphorus insecticide, induces oxidative stress in the blood serum, liver, and gill tissues of rainbow trout (*Oncorhynchus mykiss*). The addition of 0.5% curcumin was able to attenuate these negative consequences, as evidenced by a decrease in total oxidant status and oxidative stress index, while the total antioxidant capacity increased [113]. 

Alternatively, bacterial infections such as *Aeromonas* spp. can also induce oxidative stress in aquatic animals. A study by Mahmoud et al. [114] demonstrated that the addition of curcumin to the diet could potentially improve immune function, antioxidant status, growth performance, feed utilization, and disease resistance in Nile tilapia (*Oreochromis niloticus*) fish. Specifically, a concentration of 50 mg/kg curcumin in the diet exhibited superior antioxidant capability compared to other concentrations. 

Furthermore, marine fish larvae exhibit higher metabolic rates and oxygen consumption levels compared to juveniles and adults. As a result, this developmental stage in fish is also associated with oxidative stress [115]. Xavier et al. [116,117] suggested that the inclusion of curcumin in the diet of gilthead seabream postlarvae could improve their health status, enhance robustness, and increase their total antioxidant capacity and digestive capacity, while reducing protein oxidative damage and oxidative stress throughout ontogeny.

**Table 5:** Regulatory effects of curcumin on oxidative stress in aquatic animals.

Animal Category	Experimental Design	Findings (Comparison to Negative Control)	Source
Spotted seabass (*Lateolabrax maculatus*) juveniles	A total of 180 fish were divided into 3 groups: Con, TAE2 (2 g/kg diet turmeric aqueous extract), and TAE4 (4 g/kg diet turmeric aqueous extract). H_2_O_2_ (600 mM) induced oxidative stress.	Growth performance: ↑weight gain, ↔(survival, feeding rate, feed efficiency, condition factor). Hepatic antioxidant enzymes: ↑T-AOC, ↑SOD, ↑CAT, ↑GPx, ↓MDA. Serum biomarkers: ↓GPT, ↓GOT, ↓LDH. ↑the expression of Nrf2, ho-1, gcl (TAE2, TAE4). ↔keap1 expression. ↑survival (TAE4). ↑the expression of Nrf2, ho-1, gclc.	[109]
Adult zebrafish	Embryos of zebrafish were obtained by natural mating and spawning. After 7–9 h post-fertilization, embryos were transferred to a 12-well plate and divided into 4 groups (15 embryos/group): G1 (control), G2 (H_2_O_2_ 5 mM), G3 (H_2_O_2_ 5 mM + TLE 100 µg/mL), and G4 (H_2_O_2_ 5 mM + TLE 200 µg/mL).	↓the death cell ratio. ↓ROS. ↓lipid peroxidation.	[110]
Common carp (*Cyprinus carpio*) juveniles, weighed 42.3 ± 3.68 g	A total of 540 juveniles were divided into 4 groups: 0TCu (0 g turmeric/kg diet), 0TCu (0 g turmeric/kg diet) 5TCu (5 g turmeric/kg diet), 10TCu (10 g turmeric/kg diet), and 20TCu (20 g turmeric/kg diet). Experiment 1: The fish were exposed to 3.5 mg/L of ambient copper for 24 h. Experiment 2: The fish were exposed to 0.25 mg/L of ambient copper for 3 weeks.	Experiment 1: ↓mortality rate. Experiment 2: ↓cortisol, ↓glucose, ↑T4, ↑T3. ↑Lysozyme, ↑ACH50, ↑bactericidal activity. ↑SOD, ↑CAT, ↑GPx, ↓MDA. ↓TNF-α, ↓IL1-b, ↑IL-10. ↓AST, ↓ALT, ↑red blood cells, ↑hemoglobin, ↑hematocrit.	[111]
Healthy *Oreochromis niloticus*, weighed 36.05 ± 0.31 g	A total of 180 fish were divided into 4 groups: G1 (control, a basal diet), G2 (CUR 200 mg/kg diet), G3 (a basal diet containing 1% MEL), and G4 (CUR 200 mg/kg + MEL 1% diet).	Mortalities and gross changes: normal skin coloration. Growth and whole-body composition: ↑FBW, ↑WG. Hematological variables: ↑WBCs, ↑heterophils, ↑lymphocytes, ↑eosinophils, ↑monocytes. Minimized the reductions in lysozyme activity, NO, C3, IgM. Minimized the reduction of in total protein, globulin, α globulin-1, α globulin-2, γ globulin. Oxidative stress indices: ↑GPx, ↑SOD, ↑MDA. Immune-related genes: ↓TNF-α, ↓IL-1β.	[112]
Rainbow trout (*Oncorhynchus mykiss* W., 1792)	A total of 120 fish were divided into 6 groups: CON (control), CPF (0.04 mg/L CPF), CUR1 (0.5% CUR), CUR2 (1% CUR), CPF + CUR1 (0.04 mg/L CPF + 0.5% CUR), and CPF + CUR2 (0.04 mg/L CPF + 1% CUR).	Blood serum: ↓TOS, ↓OSI, ↑TAC (CFP + CUR2). Liver tissue: ↓TOS, ↓OSI, ↑TAC. Gill tissue: ↓TOS, ↓OSI (CFP + CUR), ↔TAC.	[113]
Nile tilapia fish (*Oreochromis niloticus*), weighed 2.55 ± 0.003 g	A total of 300 fish were divided into 5 groups: control, CUR50, CUR100, CUR150, CUR200 (0, 50, 100, 150, or 200 mg CUR/kg diet, respectively). Injection of both *Aeromonas hydrophila* and *Aeromonas sobria*.	↑antibacterial activity CUR50: The best value of FW, DWG, SGR, FI. Body composition: ↑crude lipid%, ↑crude protein% (max: CUR50). Oxidative status: ↑CAT (max: CUR50), ↑GSH (Max: CUR50). ↓MDA (min: CUR50), ↑Lysozyme activity (max: CUR50). ↑immunoglobin levels (max: CUR50): IgM, IgG. Intestinal microbiota: ↓coliforms, ↓*E. coli*, ↓*Aeromonas* spp. ↑survival rate (max: CUR50).	[114]
Gilthead seabream larvae of 4 days after hatching	Larvae were distributed in 9 cylindro-conical tanks (100 L) and divided into 3 microdiet groups: CLRL (control), LOW (CUR: 1.5 g/kg feed), and HIGH (CUR: 3.0 g/kg feed).	↔growth performance. Feeding Incidence: ↔LOW, ↑HIGH. Digestive enzymes: ↑trypsin, ↑chymotrypsin, ↔aminopeptidase, ↔4C-like lipase, 18C-like lipase (↓at 24 DAH, ↔at 31 DAH), alkaline phosphatase (↓at 24 DAH, ↔at 31 DAH), ↔amylase. Antioxidant status: ↑GSH, ↑TAC (24 and 31 DAH). ↓PC (10 and 31 DAH), ↓mtROS (24 and 31 DAH).	[116]
Gilthead seabream postlarvae	Postlarvae were kept in 100 L tanks, at an initial density of 2200 individuals (22 postlarvae/L). Treatment diets were divided into 3 groups: CTRL (control), LOW (curcumin: 0.8 g/kg feed), and HIGH (curcumin: 1.5 g/kg feed).	↔Growth performance. Oxidative status: ↑TAC, ↓PC, ↔the expression of antioxidant defenses (SOD1, CAT, GPx1, GPx3), ↔hsp90aa, ↑GR. Gut morphometry and function: ↔the expression of pept1 and ialp genes.	[117]

Abbreviations: ↑(increase/upregulation), ↓(decrease/downregulation), ↔(no change), CUR (curcumin), T-AOC (total antioxidant capacity), SOD (superoxide dismutase), CAT (catalase), GPx (glutathione peroxidase), MDA (malondialdehyde), LDH (lactate dehydrogenase), GOT (oxalate transaminase), GPT (pyruvate transaminase), TLE (turmeric leaf extract), AST (aspartate aminotransferase), ALT (alanine aminotransferase), MEL (melamine), GPx (glutathione peroxidase), FBW (final bodyweight), WG (weight gain), CPF (chlorpyrifos), TOS (total oxidant status), OSI (oxidative stress index), TAC (total antioxidant capacity), FW (final wet weight), DWG (daily weight gain), SGR (specific growth rate), FI (feed intake), DAH (days after hatching), GSH (glutathione), GR (glutathione reductase).

### 6.2. Ammonia Stress Management in Aquatic Animals

Similar to oxidative stress, ammonia poisoning has led to the growth of ROS levels, inflammatory cytokines, inflammatory mediators, and the expression of antioxidant enzymes, while declining cell viability and the expression of anti-apoptosis. By contrast, curcumin could improve these negative impacts on kidney macrophage of Yellow catfish *Pelteobagrus fulvidraco* related to the NF-κB/COX-2 pathway [118] (Table 6). Referring to juvenile greater amberjack (*Seriola dumerili*), the dietary curcumin supplementation suppressed stress symptoms in the liver, spleen, intestinal, and gill by upregulating the relative expression of CAT and Mn-SOD, increasing immune enzyme activity and antioxidant capacity, and significantly promoting hepatic alkaline phosphatase (ALP) and acid phosphatase (ACP) levels [12,119,120] (Table 6). 

### 6.3. Thermal Stress Management in Aquatic Animals

Thermal stress, including heat and cold stress, has a specific influence on fish physiology [121]. However, there are limited studies on the effects of curcumin in managing thermal stress in fisheries. Certainly, heat stress has been found to decrease the level of immunoglobulins, while increasing ALT and AST, which are indicators of liver injury in fish. In a study by Mahanty et al. [122], curcumin, a bioactive compound, was shown to improve thermotolerance in *Puntius sophore*. This was achieved through the upregulation of Nrf-2, SOD, CAT, GPx, and heat-shock protein (hsp) expression in the gill and liver tissues. In addition, Abdel-Ghany et al. [123] proposed that nanocurcumin, in its free-form, has a dominant role in alleviating growth performance, nonspecific immunity, stress indicators, and heat stress resistance in Nile tilapia. The recommended dose for these effects is 100 mg/kg in the diet (Table 7).

Different from heat stress, cold stress is a challenge for many fish when they suffer from a rapid decline in temperature, resulting in a cascade of physiological and behavioral responses and a more serious case of death [124]. Under low-temperature conditions, although the supplement of nanocurcumin to Nile tilapia diets has no significant changes in nutrient efficiency, hematological components, and survival rates, fish that consumed nanocurcumin diets showed an improvement in antioxidant capacity (the increase of SOD, GPx, and CAT levels) and healthier gastrointestinal microbiota [125] (Table 7).

**Table 7:** Regulatory effects of curcumin on thermal stress in aquatic animals.

Animal Category	Experimental Design	Findings (Comparison to Negative Control)	Source
Heat Stress			
Nile tilapia (*Oreochromis niloticus*), weighed 13.54 ± 0.32 g	A total 168 fish were divided into 7 groups: CON, CN50, CN100, and CN200 (nanocurcumin: 50, 100, and 200 mg/kg diet, respectively); C50, C100, and C200 (curcumin: 50, 100, and 200 mg/kg diet). Raising the water temperature from 25 to 40 °C within 3 h, and then 40 °C for 4 h.	Enhancing the growth performance (CN100, CN200). Liver enzymes activities: ↓ALT, ↓AST (CN50, CN100). ↑IgM, ↑C3 (except C50), ↑C4. ↓Cortisol (CN50, CN100). Nanocurcumin is more effective than its free form.	[123]
*Puntius sophore*, a minor carp of the family Cyprinidae	Fish were divided into 4 groups (40 fish/group): A (basal diet), B, C, and D (0.5, 1, and 1.5% curcumin-supplemented feed, respectively → highest CT max value: D. → A and D: heat shocked. → Gene expression analysis in 3 groups: BD (basal diet), BD + HS (basal diet + heat shocked), and 1.5%CUR + HS (1.5% curcumin supplemented + heat shocked).	Gene expression in liver tissues: ↑Nrf2, keap-1: very low, ↑hsp70, ↑hsp110, ↑[hsp27, hsp60] insignificant, hsp90: very low. ↑SOD, ↑CAT, ↔GPx. Gene expression in gill tissues: ↑Nrf2, ↔keap-1, ↑[hsp60, hsp70, hsp90, hsp110], ↑SOD, ↑CAT, ↑GPx. Network analysis: direct binding and interaction between all the hsp, CAT has nonspecific interaction with all of the hsp, nrf-2, and keap-1. GPx has no direct interaction with any genes.	[122]
Cold Stress			
The Nile tilapia juveniles, weighed 4.39 ± 0.08 g/fish	A total of 225 fish were divided into 5 groups: T1 (control), T2 (50 ppm nanocurcumin), T3 (100 ppm nanocurcumin), T4 (150 ppm nanocurcumin), and T5 (200 ppm nanocurcumin). Under chronic low temperature (21.02 ± 0.11 °C).	↔Digestive enzymes Blood health: Ht, Hb, RBCs, and WBCs exposed insignificant alteration. Serum biochemists: ↔[triglyceride, ALT, AST], ↑total protein, ↓[glucose, cortisol, total cholesterol]. ↑lysozyme, bactericidal activities. Antioxidant potency: ↑SOD, ↑GPx, ↑CAT. Gastrointestinal microflora: ↔[TBC, TYMC], ↓coliform.	[125]

Abbreviations: ↑(increase/upregulation), ↓(decrease/downregulation), ↔(no change), Ht (hematocrit), Hb (hemoglobin), RBCs (red blood cells), WBCs (white blood cells), ALT (alanine transaminase), AST (aspartate transaminase), TBC (total bacterial count), TYMC (total yeast and molds count), SOD (superoxide dismutase), CAT (catalase), GPx (glutathione peroxidase).

### 6.4. Stress Due to Stocking Densities in Aquatic Animals

Besides the stressors presented above, high stocking density is a cause of chronic stress in farmed fish [126]. In particular, as illustrated in the study of Akdemir et al. [127] about rainbow trout, *Oncorhynchus mykiss* (Walbaum), the figures of MDA (in serum and the liver), HSP70, HO-1, and NF-Κb levels in high stocking density (100 kg/m^3^) were higher than those in low stocking density (20 kg/m^3^). On the contrary, after the addition of curcumin to the diet, these harmful effects could be attenuated [127]. 

## 7. Conclusions and Future Outlook

**Figure 4:**
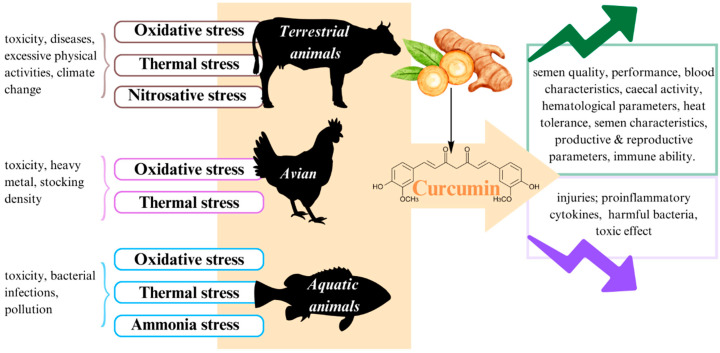
A schematic diagram on curcumin in animal stress management.

In brief, as previously clarified, stress is attributed to impaired productivity performance in livestock, poultry, and fisheries industries. To solve this problem, it is important to find a safe, nontoxic, and inexpensive measure. The application of antioxidant components such as curcumin in stress management is a potential measure. Indeed, according to studies during the last decade, curcumin has more outstanding functionality in improving the harmful effects of stress on animals than other natural compounds (Figure 4). Nonetheless, curcumin also has some disadvantages because of its low bioavailability. Furthermore, curcumin in nanoformulation seems to be dominant compared to its usual form if used in appropriate concentrations. Consequently, it is necessary to carry out further studies about the use of nanocurcumin with a suitable dose to effectively deliver it to the target organs of animals.

## Figures and Tables

**Table 1:** Regulatory effects of curcumin on oxidative stress in terrestrial animals.

Animal Category	Experimental Design	Findings (Comparison to Negative Control)	Source
Toxicity			
Healthy New Zealand rabbits, aged 6–8 weeks, weighed 1–1.5 kg	A total of 35 male rabbits were divided into 5 groups: control, AFB1, AFB1 + CUR (15 mg/kg b.w.), AFB1 + ZnO-NPs (25 µg/kg b.w.), and AFB1 + ZnO-NPs (50 µg/kg b.w.). AFB1 were orally given at a dose of 50 µg dissolved in 0.5 mL of olive oil/animal.	Antioxidant parameters: ↓NO, ↓MDA ↑GSH, ↑SOD, ↑CAT. Serum biochemical parameters: ↓AST, ↓ALT, ↓cholesterol, ↓triglyceride. Genotoxic effect on hepatic cells and renal cells.	[32]
BALB/c mice, aged 5 weeks age, weighed 20–22 g	A total of 50 male mice were divided into 5 groups: control, CUR200 (200 mg/kg b.w. CUR), AFB1 (750 µg/kg b.w. AFB1), AFB1 + CUR100 (750 µg/kg b.w. AFB1 + 100 mg/kg b.w. CUR), and AFB1 + CUR200 (750 µg/kg b.w. AFB1 + 200 mg/kg b.w. CUR). These compounds were administered in olive oil (0.2 mL) as a vehicle via oral gavage.	↑bodyweight. Renal dysfunction: ↓BUN, ↓CREA, ↓UA. Pathological slices: no significant lesions (G5). Kidney oxidative damage: ↓MDA, ↓H_2_O_2_, ↑T-AOC, ↑CAT, ↑SOD, ↑GSH. Keap1-Nrf2 signaling pathway: ↑[CAT, SOD1, NQO1, GCLC], ↑the protein expression level of Nrf2 (G5), ↓Keap1, ↓the apoptosis rate of kidney cells, ↓[Bax, caspase-9, caspase-3], ↑Bcl-2.	[34]
Wistar albino rats, weighed 34–36 g	A total of 38 male rats were divided into 5 groups: G1 (control), G2 (1 mL 10% DMSO), G3 (300 mg/kg CUR), G4 (250 µg/kg AFB1), and G5 (250 µg/kg AFB1 + 300 mg/kg CUR). AFB1 and CUR were dissolved in 10% DMSO.	↑bodyweight average after 60 days. Kidney function: ↓BUN, ↓uric acid, ↓creatinine.	[33]
Wistar albino rats, aged 12 weeks, weighed 160–180 g	A total of 40 adult male rats were divided into 4 groups and treated orally: control (distilled water), CUR + VitC (200 mg/kg b.w. CUR + 100 mg/kg b.w. VitC), CPM (200 mg/kg b.w.), and CPM + CUR + VitC (200 mg/kg b.w. CPM + 200 mg/kg b.w. CUR + 100 mg/kg b.w. VitC). Curcumin was obtained from the powder of *Curcuma longa*.	Serum: ↑ATPase, ↓MDA, ↓PC, ↑GSH, ↑SH pt, ↑CAT, ↑SOD, and ↓GST. Brain: ↑ATPase, ↑MDA, ↓PC, ↑GSH, ↑CAT, ↔SOD, and ↓GST. Liver: ↑ATPase, ↓MDA, ↔PC, ↑GSH, ↑CAT, ↓SOD, and ↑GST.	[35]
Kunming mice, aged 9 weeks, weighed 45 ± 2 g	A total of 48 male mice divided into 4 groups: control group (distilled water 2 mg/kg), Cd group (CdCl_2_ 2 mg/kg), CUR group (CUR 50 mg/kg), and Cd + CUR group (CdCl_2_ 2 mg/kg + CUR 50 mg/kg). After the mice were injected with CUR solution for 4 h, they were injected with CdCl_2_ solution.	↑sperm motility, ↑sperm concentration, ↓abnormal sperm rate, ↑serum testosterone level. ↑GSH-Px, ↑GSH, ↑SOD, ↓MDA. ↑Spermatogenic cells, mature spermatozoa in the testicular seminiferous tubules. ↑the spermatogenic cells, mature spermatozoa ↑mRNA and proteins expression [Nrf2, GSH- Px, γ- GCS].	[36]
Wistar rats, weighed 230 ± 20 g, aged 8 weeks old	A total of 70 adult male rats were divided into 7 groups: control, Ni50 (50 mg/kg NiNPs), Ni50 + GA150 (50 mg/kg NiNPs + 150 mg/kg GA), Ni50 + GA300 (50 mg/kg NiNPs + 300 mg/kg GA), Ni50 + CUR100 (50 mg/kg NiNPs + 100 mg/kg CUR), Ni50 + CUR300 (50 mg/kg NiNPs + 100 mg/kg CUR), and Ni50 + GA300 + CUR300 (50 mg/kg NiNPs + 300 mg/kg GA + 300 mg/kg CUR).	↓Glucose, ↓triglyceride, ↓cholesterol, ↓LDL, ↓HDL. ↓ALT, ↓AST, ↓ALP, ↓bilirubin, ↑albumin, ↑total protein. ↓BUN and ↓creatinine. ↑LH, ↑FSH, ↑dihydrotestosterone, ↑testosterone.	[37]
Male rats, weighed 220–250 g, aged 10 weeks old	A total of 35 male rats were divided into 5 groups: G1 (control), G2 (As 10 mg/L). G3 (As 10 mg/L + CUR 80 mg/kg), G4 (As 10 mg/L + CUR 160 mg/kg), and G5 (As 10 mg/L + CUR 240 mg/kg). Curcumin was extracted from rhizomes of turmeric by a Soxhlet apparatus.	Liver function: ↓ALT, ↓AST, ↓ALP. Kidney function: ↓total bilirubin, ↓urea, ↓creatinine. Serum lipid profile: ↓total cholesterol contents, ↓total triglyceride contents, ↑HDL, ↓LDL. Antioxidant markers (liver and kidney): ↓MDA, ↑SOD, ↑CAT, ↑GPx, ↑GR.	[38]
*Sarcoptes*-infested rabbits, aged 60 days, weighed 869.5 ± 94.27 g	A total of 83 rabbits were divided into 3 groups: G1 (control), G2 (IVM 0.2 mg/kg b.w.), G3 (IVM 0.2 g/kg b.w. + TE 1 mg/kg diet), and G4 (IVM 0.2 g/kg b.w. + TE 2 mg/kg diet). The aqueous extract of turmeric was obtained from the rhizome of *Curcuma longa*.	Growth performance: ↑final weight, ↑wt. gain, ↓mortality rate. Improved nutrient digestibility of DM, CP, NDF, ADF. Day 30: ↓ALT, ↓AST. ↓TBARS (Day 30), ↑T-AOC, ↑SOD (Day 30), ↑GSH-Px.	[39]
Adult Holstein Friesian breeding bulls	Ejaculates were collected from 5 adults bulls and divided into 10 groups: -Groups untreated with FeAA: G1 (2.9% SC control), G2, G3, G4, and G5 (2.9%SC + 5, 10, 25, and 50 µmol/L CUR, respectively). -Groups treated with FeAA (150 µmol/L FeSO_4_ + 750 µmol/L ascorbic acid): G6 (FeAA control), G7, G8, G9, and G10 (FeAA + 5, 10, 25, and 50 µmol/L CUR, respectively).	↑MOT, ↑PROG. ↑mitochondrial activity. ↓ROS, ↓superoxide generation (25, 50 µmol/L CUR). ↑SOD, ↑CAT (FeAA treatment), ↑GSH. ↓MDA (FeAA treatment). ↔MDA (FeAA untreated). ↑GPx.	[40]
RAW264.7 cells	Cells were stimulated with curcumin (0, 5, 10, and 20 μM) for 20 h. The positive control group and the curcumin-treated groups were then exposed to H_2_O_2_ (500 μM) for 4 h or 8 h, respectively.	↑cell viability (5 μM). ↑CAT, ↑SOD, ↑GSH-Px. ↓MDA, ↓ROS (5 and 10 μM). Protecting cells from apoptosis. Active Nrf2 (5 and 10 μM). ↑the expression of hemoxygenase-1. ↑the expression of glutamate–cysteine ligase. ↓transcription of glutamate–cysteine ligase.	[41]
Inflammation and Diseases			
Buffalo mammary epithelial cells (BuMECs)	Cells were seeded in 6-well plates in DMEM and divided into 4 groups: G1 (control), G2 (LPS: 5 µg/mL, 6 h), G3 (HECl 50 µg/mL 24 h + LPS 5 µg/mL 6 h), and G4 (EECl 50 µg/mL 24 h + LPS 5 µg/mL 6 h). The powder of the rhizome of *C. longa* was dissolved in the two solvents (hexane and ethanol).	The highest DPPH at the doses of 50 and 100 µg/mL. ↓TLR4 expression (G3 and G4) Inflammatory gene expression - G3: ↓TNFα, ↓IL-6, ↓NFκB - G4: ↓TNFα, ↔IL-6, ↓NFκB ↑Nrf2 expression (G3 and G4).	[42]
Swiss albino mice, weighed 25–35 g	A total 75 adult female mice were divided into 5 groups: G1 (healthy control), G2 (*S. aureus* infected), G3 (Vehicle control: 0.1 mL/10 g b.w. gum acacia), G4 (CUR 100 mg/kg b.w.), and G5 (CUR-NP: 10 mg/kg b.w. CUR in nanoparticle-encapsulated form) for 24 h, 48 h, and 72 h.	↔lymphocytes, ↓neutrophils ↓LPO, ↑SOD (24 h, 48 h), ↓SOD (72 h) ↓CAT (G4-24 h, 72 h, G5-48 h, 72 h), ↑CAT (G5-24 h), ↓GST (24 h, G5-48 h), ↑GST (72 h, G4-48 h). ↓congestion, inflammatory cellular infiltration, and edema. Normal secretion of milk.	[43]
Crossbreed calves, weighed 201 ± 14 kg, aged 8 months	A total of 331 crossbreed calves were divided into 4 groups: control, MON (monensin: 22 mg/kg d.m.), TUR100, and TUR200 (100 and 200 mg/kg d.m. TUR, respectively). TUR extract, 95%. Treatment: bovine respiratory disease (BRD).	Decreased the number of calves that required a third medication for BRD compared with MON.	[44]
Weaned			
Wuzhishan piglets, aged 35 days, weighed 3.54 ± 0.28 kg	A total of 50 weaned piglets were divided into 5 groups: G1 (control), G2 (50 mg/kg PIP), G3 (200 mg/kg CUR), G4 (200 mg/kg CUR + 50 mg/kg PIP), and G5 (300 mg/kg CUR).	↔initial weight, ↔final weight F/G (G4 and G5): lower than others. In the jejunal and ileum mucosa: ↔IL-1β, ↔TNF- α, ↔IL-6, ↔IL-10. G4 and G5: ↑SOD, ↑GSH-Px, ↓MDA, ↔T-AOC.	[45]
Buffalo granulosa cells (GCs)	GCs were divided into 6 groups: G1 (control), G2 (DMSO), G3 (1 μM CUR), G4 (2.5 μM CUR), G5 (5 μM CUR), and G6 (10 μM CUR) for 24 h and 48 h at 37 °C.	↓viability (in vitro). ↑mitochondrial activity (G4, G5). ↑ROS (G6). Enzyme activity (CAT, SOD, GSH, DPPH): 24 h (↑G5), 48 h (↓all groups).	[46]
Excessive Physical Activity			
Wistar rats, aged 12 weeks	A total of 48 male rats were divided into 6 groups: G1 (AIN-93M: standard diet), G2 (AIN-93M + ET), G3 (WPC + CUR), G4 (WPC + CUR + ET), G5 (CUR), and G6 (CUR + ET). WPC (44.0 g/kg diet), CUR (1.2 g/kg diet).	Gene expression: ↓TNF-α, ↓IL-6, ↑IL-10. Biomarker for oxidative stress: ↓MDA, ↓PC, ↓NO (G4). Antioxidant enzymes: ↑CAT (ET), ↑SOD, ↑GST (G5).	[47]
Growth Performance			
Wistar rats, weighed 150–230 g	A total of 18 male rats were divided into 3 groups: control, T1 (2.5% cinnamon + turmeric extract), and T2 (5% cinnamon + turmeric extract). The extract of cinnamon and turmeric mixture was incorporated at 2.5 and 5% concentration into powdered diet.	% weight gain: T1 (31.8%), control (38.17%), and T2 (38.24%). Organ weight: ↑liver, ↔kidney, ↔heart. Enzyme assays: ↑CAT (in liver), ↓SOD (in liver and muscles_T2), ↔GST, ↔LDH, ↔MDH, ↔AST, ↔ALT. ↔Blood parameters (Hb, RCB, PCV, MCV, MHC, MCHC, PLT). Serum liquid profile: The animals in T2 have high cholesterol.	[48]

Abbreviations: ↑(increase/upregulation), ↓(decrease/downregulation), ↔(no change), AFB1 (aflatoxin B1), NO (nitric oxide), MDA (malonaldehyde), GSH (glutathione), SOD (superoxide dismutase), CAT (catalase), AST (aspartate amino-transferase), ALT (alanine aminotransferase), ZnO-NPs (zinc oxide nanoparticles), CUR (curcumin), CUR-NP (nanoparticle-encapsulated curcumin), TUR (Turmeric), d.m. (dry matter), b.w. (bodyweight), Hb (hemoglobin), RCB (red blood cell), PCV (packed cell volume), MCV (Mean corpuscular volume), MCHC (mean corpuscular hemoglobin concentration), PLT (platelet count), GST (glutathione-s-transferase), HECl (the hexanic extract of *C. longa*), EECl (the ethanolic extract of *C. longa*), TLR (toll-like receptor), TNF (tumor necrosis factor), IL (interleukin), NFκB (Nuclear Factor Kappa B), CPM (cypermethrin), VitC (vitamin C), PC (protein carbonyl), WPC (whey protein concentrate), ET (exhaustion test), T-AOC (total antioxidant capacity), FeAA (ferrous ascorbate), MOT (percentage of motile spermatozoa), PROG (percentage of progressive motile spermatozoa), GSH-Px (glutathione peroxidase), γ-GCS (γ-glutamylcysteine synthetase), DMSO (dimethyl sulfoxide), DPPH (1,1-diphenyl-2-picrylhydrazyl), PIP (piperine), F/G (the ratio of feed to gain), T-AOC (total antioxidant capacity), IVM (ivermectin), CP (crude protein), NDF (neutral detergent fiber), ADF (acid detergent fiber), TE (turmeric extract), TBARS (thiobarbituric acid reactive substance), LPO (lipid peroxidation), BUN (urea nitrogen), CREA (creatinine), UA (uric acid), NiNPs (nickel nanoparticles), GA (gallic acid), HDL (high- density lipoprotein), LDL (low-density lipoprotein), SC (sodium citrate), LDH (lactate dehydrogenase).

**Table 6:** Regulatory effects of curcumin on ammonia stress in aquatic animals.

Animal Category	Experimental Design	Findings (Comparison to Negative Control)	Source
Yellow catfish (Pelteobagrus fulvidraco). Weighted 100 ± 50 g	The kidney cells of fish were divided into 6 groups: CON (control), AM (ammonia, 0.23 mg/L), CUR (curcumin, 45 μmol/L), 5A (ammonia + curcumin 5 μmol/L), 25A (ammonia + curcumin 25 μmol/L), and 45A (ammonia + curcumin 45 μmol/L).	↑cell viability, ↓ROS. ↓mRNA levels of SOD and GPx genes. ↓expression of IL-1, IL-6, NF-κB, TNF-α, COX-2 genes. ↑expression of Arg-1 and Bcl-2. ↓number of apoptotic cells.	[118]
The wild-caught juvenile of the greater amberjack (*Seriola dumerili*)	A total of 135 juveniles were divided into 3 groups: CUR0%, CUR0.01% (100 mg/kg curcumin), and CUR0.02% (200 mg/kg curcumin). Ammonia challenge: NH_4_Cl (1 g/L farming environment).	Antioxidant capacity of the liver: the relative expression ↑CAT (CUR0.02%) after recovery, ↓GSH-Px, ↓GR, ↓Keap1, ↔Mn-SOD. Enzyme activity (SOD, GSH): ↓[after challenge], ↑[after recovery]. Antioxidant capacity of the spleen: the relative expression CAT [↑after challenge, ↓after recover in CUR0.01%], ↓GR, ↑Keap1, ↑Mn-SOD. Enzyme activity: ↑SOD, ↑GSH.	[119]
The greater amberjack (*Seriola dumerili*), weighed 151.44 ± 7.16 g	A total of 135 juveniles were divided into 3 groups: CUR0%, CUR0.01% (100 mg/kg curcumin), and CUR0.02% (200 mg/kg curcumin). Ammonia challenge: NH_4_Cl (1 g/L farming environment).	Survival and growth performance: ↑final bodyweight, ↑weight gain, ↑survival rate (CUR0.01%). Intestinal histology structure: ↑fold height (CUR0.01%), ↑muscular thickness, ↑enterocyte height. Intestinal immune enzyme activity: ↑ALP, ↑ACP, ↑LZM (CUR0.01%). Intestinal immune gene expression: ↓[C3, C4, IgT, NF-κB1], ↔[Hepc, IL-1β, TGF-β], ↑[IFN-γ, IL-10, TNF-α, MX]_CUR0.01%], ↓Il-8_CUR0.01%. ↑activity of antioxidant enzymes in the gill (SOD, GSH-Px) Relative expression of immune-related genes in the gill: ↓Keap1, ↑Hsp70 (CUR0.01%), ↑Cu-SOD, ↑GSH-Px.	[120]
Greater amberjack (Seriola dumerili), initial weight: 100.90 ± 0.03 g	A total of 225 fish were divided into 3 groups: CON (control), CUR75 (75 mg/kg curcumin), and CUR150 (150 mg/kg curcumin). Ammonium chloride (NH_4_Cl) was used as an ammonia source.	Intestinal and hepatic: ↑ALP, ↑ACP, ↑SOD, ↑T-AOC, ↓GSH, ↓GSH-Px. Liver, spleen, head kidney, and brain tissues after post-recovery: ↑[SOD, T-AOC, GSH, GSH-Px, CAT], ↓MDA.	[12]

Abbreviations: ↑(increase/upregulation), ↓(decrease/downregulation), ↔(no change), CUR (curcumin), SOD (superoxide dismutase), GPx (glutathione peroxidase), GSH-Px (Plasma glutathione peroxidase), GR (glutathione reductase), ALP (alkaline phosphatase), ACP (acid phosphatase), LZM (lysozyme), T-AOC (total antioxidant capacity), GSH (glutathione), CAT (catalase), MDA (malonaldehyde).

## Data Availability

All data are reported in this article.
